# A Droplet‐Microarray Platform for Multiplex Profiling of Breast Cancer Exosome Subtypes in Patients’ Blood Plasma Samples

**DOI:** 10.1002/smll.202513591

**Published:** 2026-07-06

**Authors:** Yi Liu, Ruolin Zhang, Jialei Song, Meng Xiao, Yue Meng, Zhiyang Li, Jingyu Shi, Yunyan Tai, Bing Gu, Mo Yang

**Affiliations:** ^1^ Department of Biomedical Engineering The Hong Kong Polytechnic University Kowloon Hong Kong SAR P. R. China; ^2^ The Hong Kong Polytechnic University Shenzhen Research Institute Shenzhen P. R. China; ^3^ Laboratory Medicine Guangdong Provincial People's Hospital (Guangdong Academy of Medical Sciences) Southern Medical University Guangzhou Guangdong P. R. China; ^4^ Department of Clinical Laboratory Medicine Nanjing Drum Tower Hospital Clinical College of Jiangsu University Nanjing Jiangsu P. R. China; ^5^ Department of Oncology Renmin Hospital Hubei University of Medicine Shiyan Hubei P. R. China; ^6^ Joint Research Center of Biosensing and Precision Theranostics The Hong Kong Polytechnic University Kowloon Hong Kong SAR P. R. China; ^7^ Research Centre for Nanoscience and Nanotechnology The Hong Kong Polytechnic University Kowloon Hong Kong SAR P. R. China

**Keywords:** breast cancer subtypes, exosomes, FRET, multiplex detection

## Abstract

Breast cancer is highly heterogeneous, with distinct subtypes—luminal A, HER2‐positive, and triple‐negative—each exhibiting unique behaviors and treatment responses. Accurate identification of these subtypes is essential for guiding personalized therapies and improving outcomes. Exosomes, small extracellular vesicles secreted by tumor cells, have emerged as critical biomarkers for cancer diagnostics due to their pivotal role in intercellular communication and their rich molecular cargo reflective of cell origin. However, current exosome‐based biosensors struggle to differentiate between breast cancer subtypes, limiting their effectiveness in precision medicine. To address this challenge, we have developed an innovative droplet‐microarray platform integrated with a DNase I‐assisted MoS_2_ FRET aptasensor for multiplexed profiling of breast cancer exosome subtypes (luminal A, HER2‐positive, and triple‐negative). This platform targets three key protein biomarkers—HER2, MUC1, and CD44—leveraging the high specificity of aptamers and the sensitivity of DNase I‐assisted signal amplification. Our approach enables high‐resolution differentiation of breast tumor exosome profiles with an impressive limit of detection (LOD) of approximately 10^2^ particles/mL. Application of this aptasensor to clinical samples demonstrated excellent predictive accuracy for breast cancer subtype discrimination (luminal A: AUC = 0.95; HER2‐positive: AUC = 0.99; triple‐negative: AUC = 0.90). By enabling precise identification of breast cancer subtypes, our approach holds promise for enhancing diagnostic accuracy and guiding personalized treatment strategies. This advancement represents a significant step forward in the application of exosome‐based diagnostics in precision oncology.

## Graphical abstract

This study presents a droplet‐microarray platform integrated with a DNase I‐assisted MoS_2_ FRET aptasensor for multiplexed profiling of breast cancer subtypes’ exosomes from patients’ plasma. By targeting HER2, MUC1, and CD44, the system enables sensitive detection and accurate discrimination of luminal A, HER2‐positive, and triple‐negative subtypes, supporting improved precision diagnostics and personalized treatment strategies.

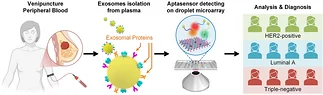

## 1 Introduction

Breast cancer is a heterogeneous neoplasm with a high incidence among females worldwide, characterized by its varied subtypes, each with distinct metastatic patterns and survival outcomes [1]. The primary subtypes, such as triple‐negative, HER2‐positive, and luminal A, exhibit unique pathological features and require tailored treatment approaches [2, 3]. Early detection and accurate diagnosis of these specific subtypes are crucial for effective clinical intervention and improving cure rates. Despite the critical importance of early detection and accurate diagnosis of breast cancer subtypes, current diagnostic methods face significant limitations in differentiating these subtypes rapidly and non‐invasively. Conventional imaging techniques, such as x‐ray mammography [4], ultrasonography [5], computed tomography (CT) [6], and magnetic resonance imaging (MRI) [7], are primarily designed to identify the presence of tumors rather than to characterize their specific subtypes. Furthermore, they may lack the sensitivity required to detect subtle molecular differences between subtypes, which are essential for precision therapy.

Recently, liquid biopsy techniques based on the detection of cancer biomarkers, including circulating tumor DNA (ctDNA) [8, 9], circulating tumor cells (CTCs) [10, 11, 12], and exosomes [13, 14, 15] in circulating body fluids for cancer early diagnostics and prognostics, are emerging as new tools for tumor precision diagnostics. Among them, exosomes are small nano‐sized extracellular vesicles with a size from 30 to 200 nm in diameter that are produced and released by cancer cells of the original tumor and then released in the circulating system. Exosomes inherit molecular characteristics from their parental cells and are readily found in body fluids such as urine and blood [16, 17], making them ideal biomarkers for convenient liquid biopsy and the diagnosis of heterogeneous tumors. Current exosome detection approaches, such as ELISA [18], flow cytometry [19], and Western blotting [20], require long reaction times and a complex operation process, and fail to meet the requirement of rapid detection. Recently, various biosensing techniques have been developed for detecting exosomes rapidly, such as surface‐enhanced Raman scattering (SERS) [21, 22, 23], surface plasmon resonance (SPR) [24, 25, 26], electrochemiluminescence (ECL) [27, 28], and fluorescence methods [29, 30, 31]. However, most of the current biosensing strategies only make a diagnosis based on one protein biomarker, which lacks the capability to differentiate tumor subtypes. Hence, it's essential to develop an efficient exosome multiplexed detection platform that can rapidly and accurately differentiate breast tumor subtypes.

Fluorescence resonance energy transfer (FRET) based assays can transduce the near‐field interaction to a far‐field signal to detect the biological phenomena when the donor‐acceptor distance is within a close distance (1–10 nm) [32, 33, 34]. This makes the FRET technique quite suitable for exosome detection. Moreover, the recent development of multiplexed FRET technique with multi‐color fluorescent probes enables the possibility of multiple target detection [35, 36], allowing for the simultaneous detection of various exosomal protein markers. Moreover, droplet microarray technology offers a novel and efficient approach for exosome detection, leveraging the precision and scalability of microarray platforms with the compartmentalization benefits of droplet‐based systems [37, 38]. Microarrays can be designed to create an array of tiny droplets, each capable of isolating and analyzing individual exosomes or small groups of exosomes. The droplet microarray format enhances sensitivity and specificity by minimizing sample dilution and cross‐contamination, while also enabling multiplexed detection of various biomarkers within the same array. Therefore, the combination of droplet microfluidics with multiplexed FRET nanoprobes will be a promising approach for high‐throughput in situ multiplexed detection of exosomal protein biomarkers.

In this work, we developed a droplet microarray multiplexed FRET sensing platform for high‐throughput profiling of breast cancer exosome subtypes in patients’ plasma. This platform consists of droplet sensor arrays with DNase I‐assisted molybdenum disulfide (MoS_2_) FRET multiplexed aptasensor integrated on the chip. Considering the heterogenous protein expression level in different subtypes of breast cancer exosomes, this platform targets breast tumor subtype‐derived exosomes based on the profiling results of protein expression discrepancy of 3 protein biomarkers, including HER2, MUC1, and CD44. We used this platform for high‐throughput profiling of breast cancer exosome subtypes in plasma from 30 breast tumor patients within 1 h. Our droplet microarray‐based multiplexed exosome sensing platform effectively distinguishes not only between samples from healthy controls and breast cancer patients, but also accurately differentiates among the triple‐negative, HER2‐positive, and luminal A breast cancer subtypes. To assess clinical performance, we compared results from our droplet microarray multiplexed exosome sensing platform with those from standard immunohistochemistry testing. The platform demonstrated significantly improved diagnostic accuracy when profiling three exosomal protein biomarkers, compared to using a single biomarker. High predictive accuracy for breast cancer subtype discrimination was achieved (luminal A: AUC = 0.95; HER2‐positive: AUC = 0.99; triple‐negative: AUC = 0.90). These findings suggest that our droplet microarray multiplexed FRET sensing platform holds great promise as a reliable tool for the accurate diagnosis of breast tumor subtypes.

## 2 Results and Discussion

### 2.1 Mechanism of MoS_2_‐Based FRET Aptasensor for Multiplexed Detection of Breast Cancer Exosome Subtypes

**SCHEME 1:**
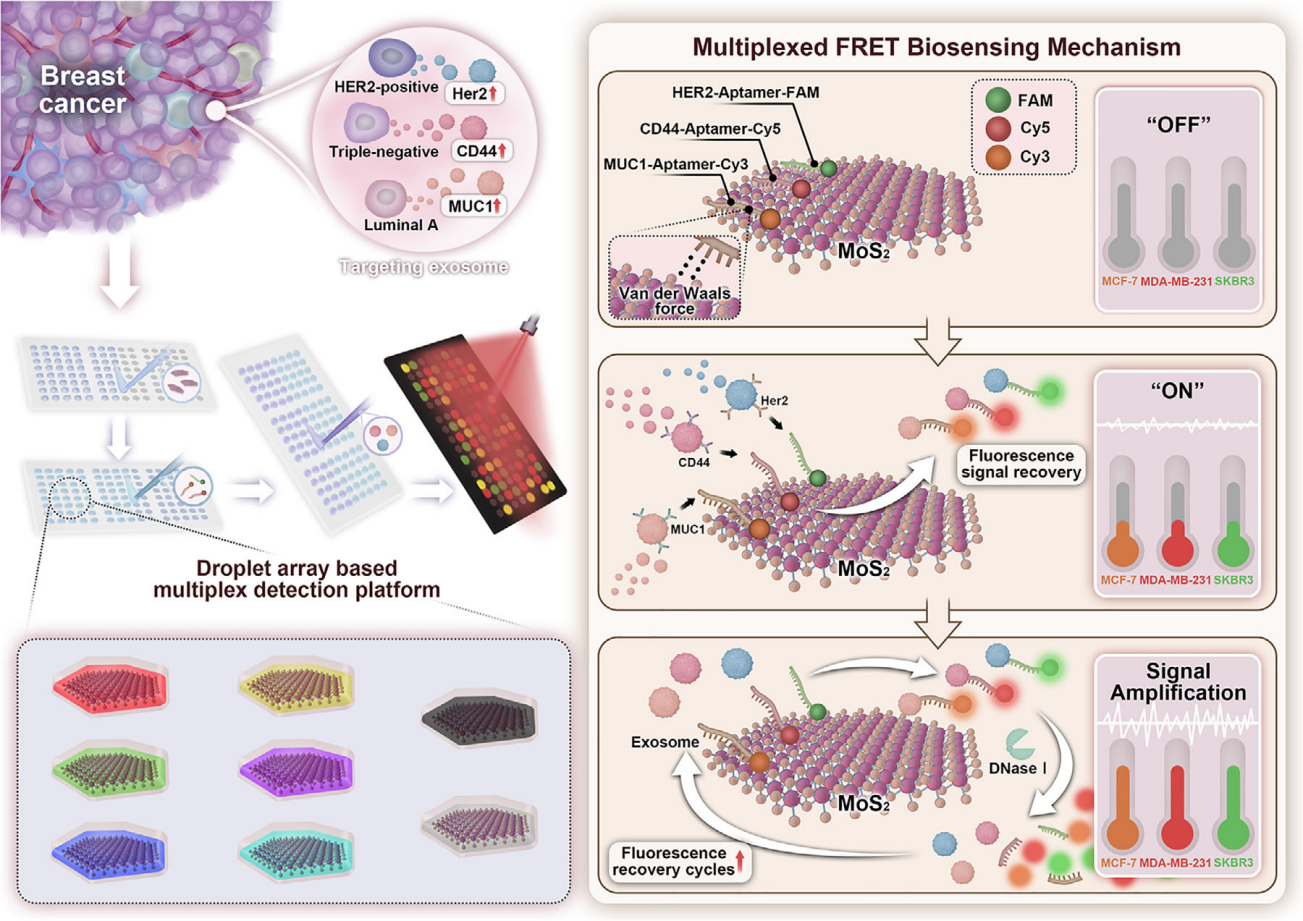
Schematic illustration of droplet array‐based multiplex detection platform for clinical breast cancer patients’ subtypes screening.

The mechanism of the MoS_2_‐based FRET aptasensor for multiplexed exosome detection is illustrated in Scheme 1. In this sensing system, MoS_2_ nanosheets serve as fluorescence quenchers by absorbing the emission from dye‐labeled aptamers, resulting in the “OFF” state of the FRET system. In the presence of exosomes, the aptamers selectively bind to their targets and detach from the surface of the MoS_2_ nanosheets, resulting in fluorescence recovery and activation of the “ON” state. The resulting change in fluorescence intensity enables sensitive detection of exosomes. To further improve detection sensitivity, DNase I endonuclease is introduced to cleave the aptamers after exosome binding, releasing the exosomes and allowing repeated cycles of aptamer‐exosome interaction. This process leads to the progressive detachment of aptamers from the MoS_2_ surface, resulting in amplified fluorescence signals. Utilizing this droplet microarray multiplex detection platform, the recovered fluorescence signals can be directly visualized under a laser scanning confocal microscope, enabling rapid and multiplexed detection of exosomes. By assigning specific fluorescence colors to each droplet, different combinations of exosome subtypes can be easily identified based on the observed fluorescence patterns within the mixture.

### 2.2 Establishment of a Droplet Microarray Chip Integrated With MoS_2_‐aptamer Multiplex FRET Biosensor

**FIGURE 1:**
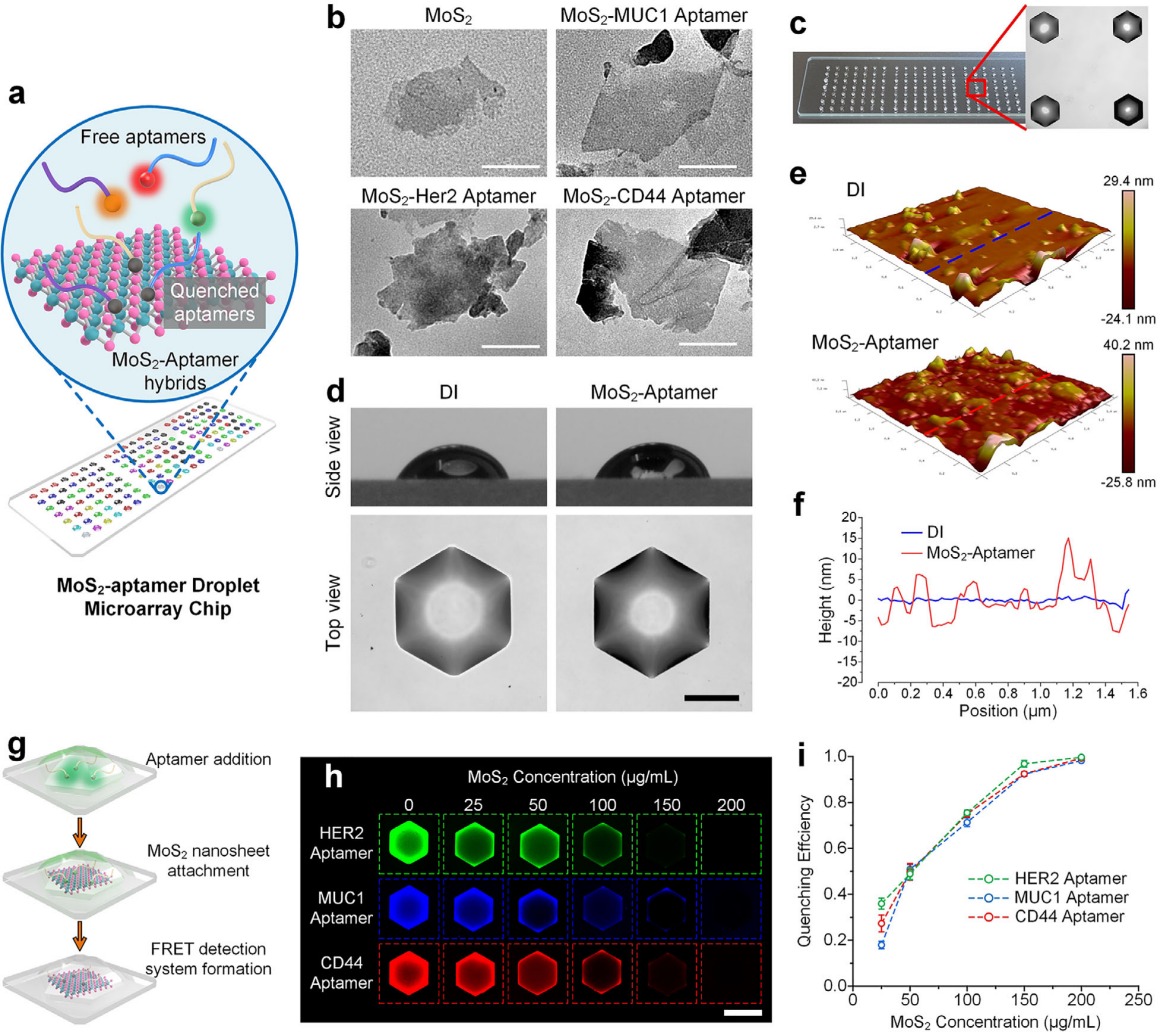
(a) Illustration of MoS_2_‐aptamer droplet microarray chip for multiplex exosome detection. (b) TEM image of MoS_2_ nanosheets, FAM‐HER2 aptamer‐conjugated MoS_2_, Cy3‐MUC1 aptamer‐conjugated MoS_2_ and Cy5‐CD44 aptamer‐conjugated MoS_2_. Scale bar: 500 nm. (c) Photograph of MoS_2_‐apatmer droplet microarray chip, inset: microscope image of a hexagon hydrophilic spot. The side length of the hexagonal pattern is 600 µm. (d) Geometries of droplets formed by continuous wetting the DI water (left) and MoS_2_‐aptamer hybrid (right) on the patterned spots. Scale bar: 500 µm. (e) AFM images of DI water‐coated surface and MoS_2_‐aptamer hybrid‐coated surface. (f) Cross‐sectional profiles corresponding to the DI water‐coated spot surface and MoS_2_‐aptamer hybrid‐coated spot surface. (g) Schematic illustration of optimizing the concentration of MoS_2_ nanosheet to obtain the optimal quenching efficiency for the FRET‐based sensing system. (h) Fluorescence images of droplets containing dye‐labelled aptamers after the addition of a series of concentrations of MoS_2_ nanosheets. Scale bar: 1 mm. (i) Quenching efficiency QE = (*I*_0_‐*I*_M_)/*I*_0_ of FAM labelled HER2‐aptamer, Cy3 labelled MUC1‐aptamer, and Cy5 labelled CD44‐aptamer at different MoS_2_ nanosheet concentrations. Data are presented as mean ± SEM (*n* = 3).

The droplet microarray chip comprises arrays of sensing spots coated with MoS_2_‐aptamer hybrids, which function as detection nanoprobes with a FRET‐based sensing mechanism (Figure 1a). We first characterized the MoS_2_‐aptamer multiplex sensing probe and the droplet microarray. The morphology of MoS_2_ nanosheets and MoS_2_‐aptamer hybrids was examined by transmission electron microscopy (TEM). As shown in Figure 1b, the MoS_2_ nanosheets exhibit a graphene‐like morphology with a large lateral surface area, which facilitates the adsorption of dye‐labeled aptamers and efficient fluorescence quenching. The hydrodynamic sizes of MoS_2_ and MoS_2_‐aptamer hybrids were further quantified by dynamic light scattering (DLS) (Figure S1a). The average size of the pure MoS_2_ nanosheets was 170.6 nm, which increased upon aptamer conjugation to 178.7 nm for MoS_2_‐HER2 aptamer, 180.7 nm for MoS_2_‐MUC1 aptamer, and 192.1 nm for MoS_2_‐CD44 aptamer. In addition, zeta potential measurements (Figure S1b) revealed that the original MoS_2_ nanosheets had a zeta potential of −11.9 mV. Following aptamer conjugation, the zeta potentials became more negative with −14.6 mV for MoS_2_‐HER2 aptamer, −16.2 mV for MoS_2_‐MUC1 aptamer, and −13.8 mV for MoS_2_‐CD44 aptamer. These results confirm the successful formation of MoS_2_‐aptamer hybrids. The spectral overlap between the absorbance of MoS_2_ and the emission spectra of each dye‐labeled aptamer satisfies the requirements for Förster resonance energy transfer (FRET) (Figure S1c). The droplet microarray chip was patterned with hexagonally hydrophilic spots, onto which MoS_2_‐aptamer hybrid detection nanoprobes were deposited for subsequent detection. The functionalization quality of the droplet microarray chip was assessed by dispensing aqueous solutions of deionized (DI) water and MoS_2_‐aptamer hybrids onto the hydrophilic spots via continuous wetting. As illustrated in Figure 1c, a total of 147 discrete nanoliter‐scale droplets were generated on the droplet microarray platform. Representative side and top view images of an individual droplet are provided in Figure 1d and Figure S3a for geometric characterization. The temporal evolution of droplet volume is depicted in Figure S3b, demonstrating that precise control over volume loss can be maintained throughout the detection period. Atomic force microscopy (AFM) was employed to evaluate the homogeneity of the sensing probe coating. As shown in Figure 1e, both DI water and MoS_2_‐aptamer hybrids exhibited uniform coatings on the hydrophilic spots. Cross‐sectional analyses revealed that both materials formed functionalized coatings with thicknesses ranging from 10 to 30 nm, which are appropriate for exosome detection (Figure 1f).

Subsequently, we then optimized the quenching efficiency of three donors (FAM‐HER2 aptamer, Cy3‐MUC1 aptamer, and Cy5‐CD44 aptamer) on the droplet microarray chip (Figure 1g). A series of concentrations of MoS_2_ (0‐200 µg/mL) and 40 nm dye‐labeled aptamers were sequentially added on the hydrophilic spots via continuous wetting. As shown in Figure 1h, the fluorescence intensity of all dye‐labeled aptamers gradually decreased with increasing MoS_2_ concentrations, and was almost completely quenched at 200 µg/mL MoS_2_. The quenching efficiency was calculated using the formula (*I*_0_‐*I*_M_)/*I*_0_, where *I*_0_ was denoted as the fluorescence intensity of the hexagon region containing only aptamers, *I*_M_ represents the fluorescence intensity of the hexagon region containing both dye‐labelled aptamers and MoS_2_. The quenching efficiency increased with higher MoS_2_ concentrations and reached a plateau at 200 µg/mL, which was therefore selected as the optimal concentration. At this concentration, the quenching efficiencies for HER2‐Aptamer‐FAM, MUC1‐Aptamer‐Cy3, and CD44‐Aptamer‐Cy5 were 99.7%, 98.2%, and 99.2%, respectively (Figure 1i). All donors exhibited quenching efficiencies greater than 98%, thereby demonstrating the feasibility of FRET‐based biosensing on the droplet microarray chip.

### 2.3 Breast Cancer Exosome Detection via Droplet Microarray Chip

**FIGURE 2:**
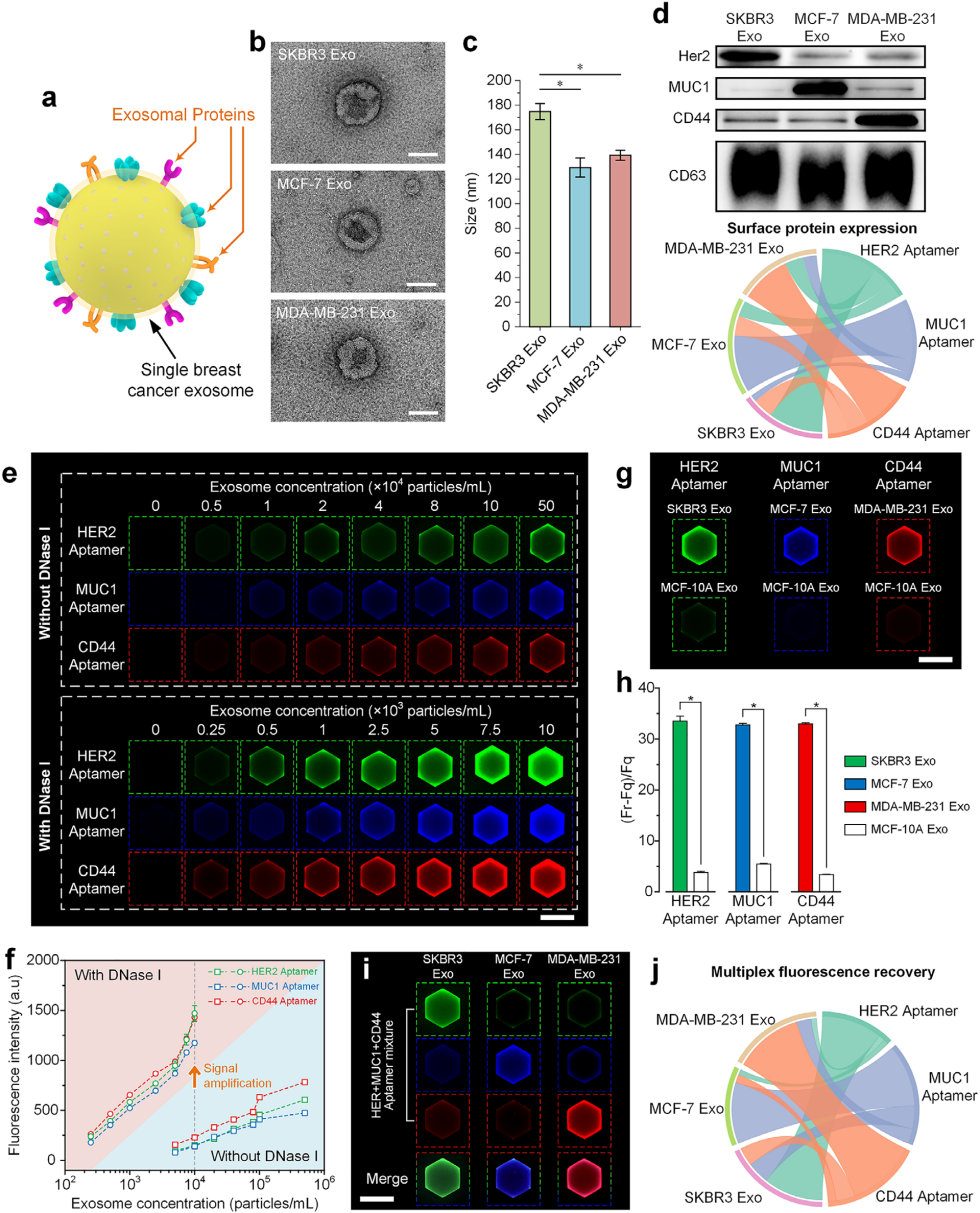
(a) Illustration of an isolated single breast cancer exosome. (b) TEM images of exosomes derived from SKBR3, MCF‐7, and MDA‐MB‐231 cells. Scale bar:100 nm. (c) The mean size analysis of exosomes derived from SKBR3, MCF‐7, and MDA‐MB‐231 cells. Data are presented as mean ±SEM. * presents *p* < 0.05 (*n* = 3). (d) Western blot analysis of HER2, MUC1, CD44, and CD63 expression levels in three breast cancer exosome subtypes, together with the assembled chord plot. (e) Fluorescence images (Scale bar: 1 mm) and (f) assembled fluorescence intensity of droplet MoS_2_‐aptamers detection platform in the presence of a series of exosome concentrations, without or with DNase I treatment Data are presented as mean ± SEM (*n* = 3). (g) Representative fluorescence images (Scale bar: 1 mm) and (h) recovery efficiency of using MoS_2_‐aptamers sensing system for detecting the corresponding breast cancer subtypes exosomes and exosomes derived from a normal breast epithelial cell line, for evaluation of detection sensitivity. Data are presented as mean ± SEM. * presents *p* < 0.05 (*n* = 3). (i) Representative fluorescence images of MoS_2_‐mixed aptamers sensing platform for detecting exosomes derived from the three breast cancer subtypes. Scale bar: 1 mm. (j) Chord diagram showing the relative fluorescence recovery ratio of MoS_2_‐mixed aptamers system in response to the three different breast cancer exosome subtypes.

Breast cancer exosomes exhibit distinct surface protein expression and phenotypic characteristics that differ depending on their cell of origin (Figure 2a). In this work, exosomes isolated from three representative breast cancer cell lines MCF‐7 (representing Luminal A subtype), MDA‐MB‐231 (representing triple‐negative subtype), and SKBR3 (representing HER2‐positive subtype), were employed for multiplex detection [39, 40]. Transmission electron microscopy (TEM) was first used to characterize the morphological features of these exosomes. As shown in Figure 2b, the breast tumor exosomes exhibited a characteristic saucer‐shaped morphology, measuring approximately 100–200 nm in diameter. Nanoparticle tracking analysis (NTA) was subsequently performed to further assess the size distribution and concentration of the exosomes. Figure S1d summarized the size distributions of breast tumor exosomes derived from MCF‐7, MDA‐MB‐231, and SKBR3 cells. The measured concentrations for all three exosome types were approximately 1 × 10^9^ particles/mL. The average diameters were 129.33 ± 7.69 nm for MCF‐7, 139.30 ± 3.99 nm for MDA‐MB‐231, and 174.80 ± 6.45 nm for SKBR3 exosomes (Figure 2c), consistent with the TEM observations. Notably, SKBR3‐derived exosomes were significantly larger on average than those from MCF‐7 and MDA‐MB‐231. Before evaluating the Western blot results, it is important to clarify the rationale for biomarker selection. To develop a noninvasive strategy for breast cancer molecular subtyping, we selected three exosomal biomarkers according to the conventional immunohistochemical classification framework used in clinical practice. MUC1 was interpreted in relation to ER/PR‐associated features [41], HER2 to indicate HER2‐enriched features [42], and CD44 to reflect biological traits associated with the triple‐negative subtype [43]. Based on this rationale, distinct subtype‐associated combinatorial expression patterns were anticipated, namely MUC1^++^/HER2^−^/CD44^−^ for Luminal A, MUC1^−^/HER2^++^/CD44^−^ for the HER2‐positive subtype, and MUC1^−^/HER2^−^/CD44^++^ for triple‐negative. Western blot analysis confirmed that CD63, a canonical exosomal marker [44], was present in exosomes from all three cell lines, supporting the successful isolation of exosomal vesicles (Figure 2d). More importantly, distinct subtype‐related protein expression patterns were observed: HER2 was highly expressed in SKBR3‐derived exosomes, MUC1 was enriched in MCF‐7‐derived exosomes, and CD44 was predominantly expressed in MDA‐MB‐231‐derived exosomes. These relative expression patterns, summarized in the chord chart in Figure 2d and bar chart in Figure S12a, are consistent with our initial hypothesis and with previous reports [45, 46, 47], indicating that the combined use of MUC1, HER2, and CD44 is reasonable for distinguishing different breast cancer subtypes at the exosomal level. Accordingly, dye‐labeled aptamers targeting HER2, MUC1, and CD44 were conjugated to MoS_2_ nanosheets to construct MoS_2_‐aptamer hybrids for breast cancer subtype exosome detection.

To detect the fluorescence signal of isolated exosomes, exosomes were applied to hydrophilic spots pre‐coated with MoS_2_‐aptamer hybrids, and fluorescence images were acquired using a laser scanning confocal microscope. Initially, a series of concentrations of isolated exosomes was added to different hydrophilic spots containing the corresponding MoS_2_‐aptamer hybrids, and representative fluorescence recovery images are shown in Figure 2e (upper panel). To further enhance the fluorescence signal, DNase I was employed to digest the aptamers, thereby releasing the exosomes previously bound to the aptamers. This process allows additional exosomes to bind to the aptamers that remain adsorbed on the MoS_2_ nanosheets, resulting in the displacement of more aptamers from the MoS_2_ surface. The DNase I‐mediated cleavage and release effect enables exosome recycling, thereby amplifying the overall fluorescence signal. The cleavage and release effect of DNase I (Figure S4a), as well as the protective capacity of MoS_2_ for adsorbed aptamers against DNase I digestion (Figure S4b), were visualized by agarose gel electrophoresis. Subsequently, a lower series of exosome concentrations was applied to another batch of hydrophilic spots, and fluorescence recovery images with amplified signals were observed; representative images are presented in the lower panel of Figure 2e. The fluorescence intensities of the detection system in the presence and absence of DNase I are summarized in Figure 2f, and the corresponding fluorescence amplification folds are shown in Figure S13. Notably, the addition of DNase I resulted in higher fluorescence recovery even at lower exosome concentrations. The recovery rates at different predefined exosome concentrations were quantified and are shown in Figure S5a–c. The recovery rate was defined as (*F*_r_‐*F*_q_)/*F*_q_, where *F*_r_ is the fluorescence intensity of MoS_2_‐aptamer hybrids with added exosomes, and *F*_q_ is the fluorescence intensity of MoS_2_‐aptamer hybrids alone. Overall, the recovery rate increased consistently with increasing logarithmic exosome concentrations.

The limit of detection (LOD) was calculated as the fluorescence intensity of the control group plus three times the standard deviation. The calculated LOD values for detecting SKBR3, MCF‐7, and MDA‐MB‐231 exosomes with DNase I treatment were 1.72 × 10^2^ particles/mL, 1.51 × 10^2^ particles/mL, and 1.92 × 10^2^ particles/mL, respectively. Similarly, the LOD values for detecting SKBR3, MCF‐7, and MDA‐MB‐231 exosomes without DNase I treatment were 5.78 × 10^3^ particles/mL, 3.62 × 10^3^ particles/mL, and 4.29 × 10^3^ particles/mL, respectively (Figure S5d–f). The specificity of the detection system was evaluated using exosomes derived from a non‐breast cancer cell line (MCF‐10A). Representative comparison images are shown in Figure 2g. The recovery rates (Figure 2h) for each aptamer in sensing the corresponding breast cancer subtype exosomes were significantly higher than those observed for non‐breast cancer exosomes. To investigate the simultaneous multiplex detection capability of the droplet MoS_2_ aptasensor, all aptamers were mixed at fixed concentrations with MoS_2_ nanosheets to sense each subtype of exosomes. Major fluorescence recovery was observed for the corresponding aptamer, while minor fluorescence recovery was detected for the other two non‐target aptamers (Figure 2i). The relative fluorescence recovery ratios are summarized in the chord chart illustrated in Figure 2j and bar chart in Figure S12b. These results are in excellent agreement with the Western blotting data presented in Figure 2d, further confirming the multiplex sensing capability of the proposed droplet MoS_2_ aptasensor. Meanwhile, this aptasensor was further verified using the well‐established fluorescence detection method of photoluminescence. The fluorescence recovery spectra, corresponding recovery efficiencies, and calculated limits of detection (LODs) are shown in Figure S6a–i. The calculated LODs for detecting SKBR3, MCF‐7, and MDA‐MB‐231 exosomes after DNase I treatment by photoluminescence were 1.47 × 10^2^, 3.95 × 10^2^, and 3.39 × 10^2^ particles/mL, respectively. These results indicate that the droplet microarray‐based aptasensor achieves detection capability comparable to that of photoluminescence, while offering a distinct advantage for simultaneous multiplex detection in a single sample.

### 2.4 Multiplex Detection of Three Subtypes of Breast Cancer Exosomes by Droplet Microarray Chip

To realize reliable multiplexed detection, it is necessary to control spectral overlap and crosstalk among the three fluorescence channels. The original emission spectra of FAM, Cy3, and Cy5 have broad tails that can overlap, so simple RGB assignment without controlling spectral spillover would be inappropriate for quantitative bioanalysis. In our system, fluorescence detection is performed on a laser‑scanning confocal microscope (SP8, Leica Microsystems) operated in multichannel acquisition mode, in which the emitted light is dispersed by a Pellin–Broca prism and directed to the detectors through tunable spectral “barriers” that define the detection windows (Figure S7a). Using this optical setup, we configured three non‑overlapping, dye‑specific windows (Figure S7b): 493–547 nm for the FAM channel (HyD 1, 488 nm excitation), 557–662 nm for the Cy3 channel (HyD 2, 552 nm excitation), and 643–778 nm for the Cy5 channel (HyD 3, 638 nm excitation). These ranges were chosen on the basis of the measured emission spectra and internal filter characteristics to truncate the broad emission tails and minimize spectral overlap at the detector level. To directly quantify crosstalk under these conditions, we imaged droplets labeled with a single aptamer–fluorophore conjugate while turning on only one excitation laser at a time, but keeping all three detection channels open simultaneously (Figure S7c). Under 488 nm excitation, a strong signal was observed exclusively in HyD 1 (FAM channel), with negligible signal in HyD 2 or HyD 3; similarly, 552 nm excitation produced signal only in the Cy3 channel, and 638 nm excitation produced signal only in the Cy5 channel. These single‑stain measurements demonstrate that, with the selected spectral windows and multichannel settings, cross‑detection between FAM, Cy3, and Cy5 is below the detection limit of our system.

On this basis, we enabled multiplex readout by assigning emission LUT colors to each aptamer–dye conjugate: HER2‑Aptamer‑FAM as green, MUC1‑Aptamer‑Cy3 as blue, and CD44‑Aptamer‑Cy5 as red, thereby establishing an RGB color model. In multiplex detection scenarios, this configuration yields seven possible color combinations: individual primary colors for single‑marker staining, secondary colors (yellow, magenta, cyan) for pairwise co‑expression, and white for triple‑positive droplets in the merged image. Prior to multiplex experiments, we performed confocal channel intensity calibration by adding equal concentrations of each dye‑labeled aptamer to the hydrophilic spots and adjusting the incident laser power and channel gain to ensure comparable fluorescence intensity across all channels, resulting in a balanced white signal in the merged image. Therefore, the RGB representations are not derived from a single broadband image with overlapping spectra, but from three individually acquired, channel‑specific images that are composited for visualization, and the displayed colors are LUT‑defined pseudo‑colors rather than the intrinsic emission colors of the fluorophores.

**FIGURE 3:**
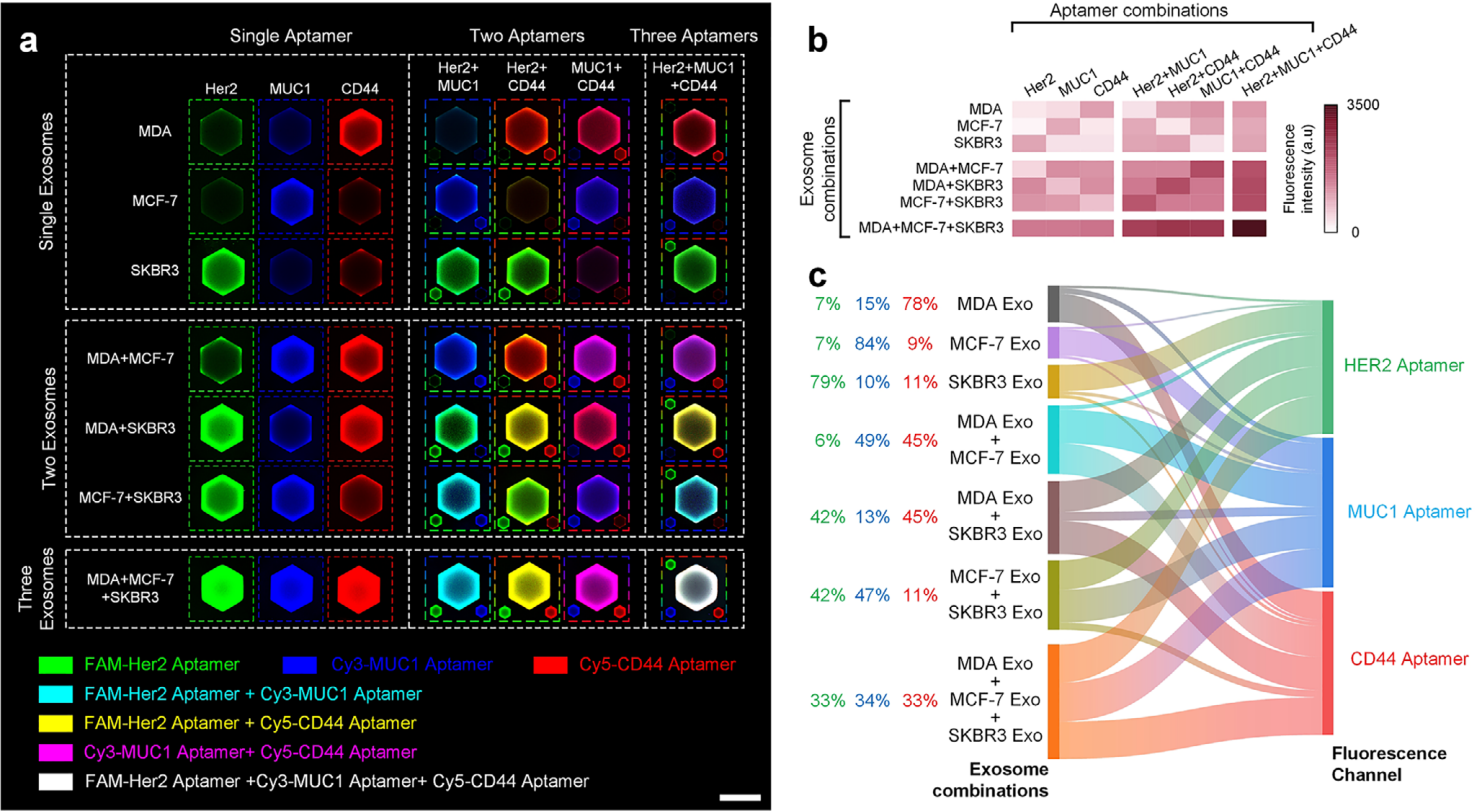
Multiplex detection of SKBR3, MCF‐7, and MDA‐MB‐231 derived exosomes using the droplet microarray chip. (a) Representative fluorescence images of the droplet arrays containing of single aptamers, two aptamers, and three aptamers for the detection of a single exosome, two exosomes, and three exosomes, respectively. Scale bar for merged images: 1 mm; the side length of the hexagonal pattern in each individual fluorescence channel is 600 µm. (b) Heatmap of assembled fluorescence intensities. (c) Sankey diagram illustrating the relationships between fluorescence intensity and exosome combinations under the three‐aptamer configuration.

The droplet microarray chip was divided into three sections, each containing a 7 × 7 array of hexagonal hydrophilic spots, allowing three independent experiments per chip. Within each array, the left three columns (C1‐C3), middle three columns (C4‐C6), and rightmost column (C7) were assigned to single‐, dual‐, and triple‐aptamer conditions, respectively. Likewise, the top three rows (R1‐R3), middle three rows (R4‐R6), and bottom row (R7) were designated for single‐, dual‐, and triple‐exosome conditions, respectively, generating 49 distinct exosome‐aptamer combinations per array. For multiplex detection, 200 µg/mL MoS_2_ was first deposited onto all detection spots by continuous wetting and dried at room temperature. Single or mixed aptamers were then added to the designated columns to hybridize with MoS_2_, followed by the addition of single or mixed exosomes to the corresponding rows. The chip was finally placed in a 100% humidity chamber on the confocal microscope stage to prevent evaporation during analysis. As shown in Figure 3a, the fluorescence images of the 7 × 7 array were assembled according to the predefined spot layout. In the single‐aptamer region, each exosome subtype produced the strongest fluorescence signal in its corresponding aptamer column: SKBR3 in the HER2‐Aptamer column (green), MCF‐7 in the MUC1‐Aptamer column (blue), and MDA‐MB‐231 in the CD44‐Aptamer column (red). The same specificity was maintained in the dual‐ and triple‐exosome regions, where strong fluorescence signals were observed whenever the corresponding exosome subtype was present in the mixture. The quantified relative fluorescence intensities are shown in Figure S8a. In the dual‐aptamer region (C4‐C6), the merged fluorescence colors accurately reflected the exosome compositions according to the predefined RGB model. In the HER2‐Aptamer + MUC1‐Aptamer column (C4), SKBR3 and MCF‐7 exosomes generated green and blue signals, respectively, while their combination produced cyan fluorescence. In the HER2‐Aptamer + CD44‐Aptamer column (C5), SKBR3 and MDA‐MB‐231 exosomes generated green and red signals, respectively, and their combination yielded yellow fluorescence. In the MUC1‐Aptamer + CD44‐Aptamer column (C6), MCF‐7 and MDA‐MB‐231 exosomes produced blue and red signals, respectively, and their combination resulted in magenta fluorescence. In mixed‐exosome regions, the observed merged colors were fully consistent with the expected fluorescence recovery patterns. The quantified results are summarized in Figure S8b. In the triple‐aptamer region (C7), MDA‐MB‐231, MCF‐7, and SKBR3 exosomes produced red, blue, and green fluorescence signals, respectively, in the single‐exosome region. Dual‐exosome combinations generated the expected magenta, yellow, and cyan signals, whereas the triple‐exosome condition produced a light white fluorescence pattern, indicating simultaneous fluorescence recovery in all three channels. The assembled fluorescence intensity heatmap of all test cases is shown in Figure 3b. Overall, the fluorescence intensity in the dual‐ and triple‐exosome regions was approximately two‐fold and three‐fold higher, respectively, than that in the single‐exosome region. Because the triple‐aptamer configuration is the final format intended for clinical detection, the fluorescence recovery characteristics of different exosome combinations were further summarized in a Sankey plot (Figure 3c). Channel analysis (Figure S8c) showed that 78% of the Cy5 fluorescence was associated with MDA‐MB‐231 exosomes, whereas 84% and 79% of the Cy3 and FAM fluorescence originated from MCF‐7 and SKBR3 exosomes, respectively, in the single‐ exosome spots. For dual‐exosome spots, two corresponding channels displayed distinct fluorescence recovery, with a combined contribution of approximately 90%. In the triple‐exosome condition, the three channels showed a nearly uniform fluorescence distribution of ∼33% each. These results confirm the feasibility of multiplex exosome detection on the droplet microarray chip through analysis of the characteristic fluorescence patterns generated by specific aptamer configurations.

### 2.5 Development and Analytical Evaluation of a Droplet Microarray Chip‐Based Assay for Clinical Samples

**FIGURE 4:**
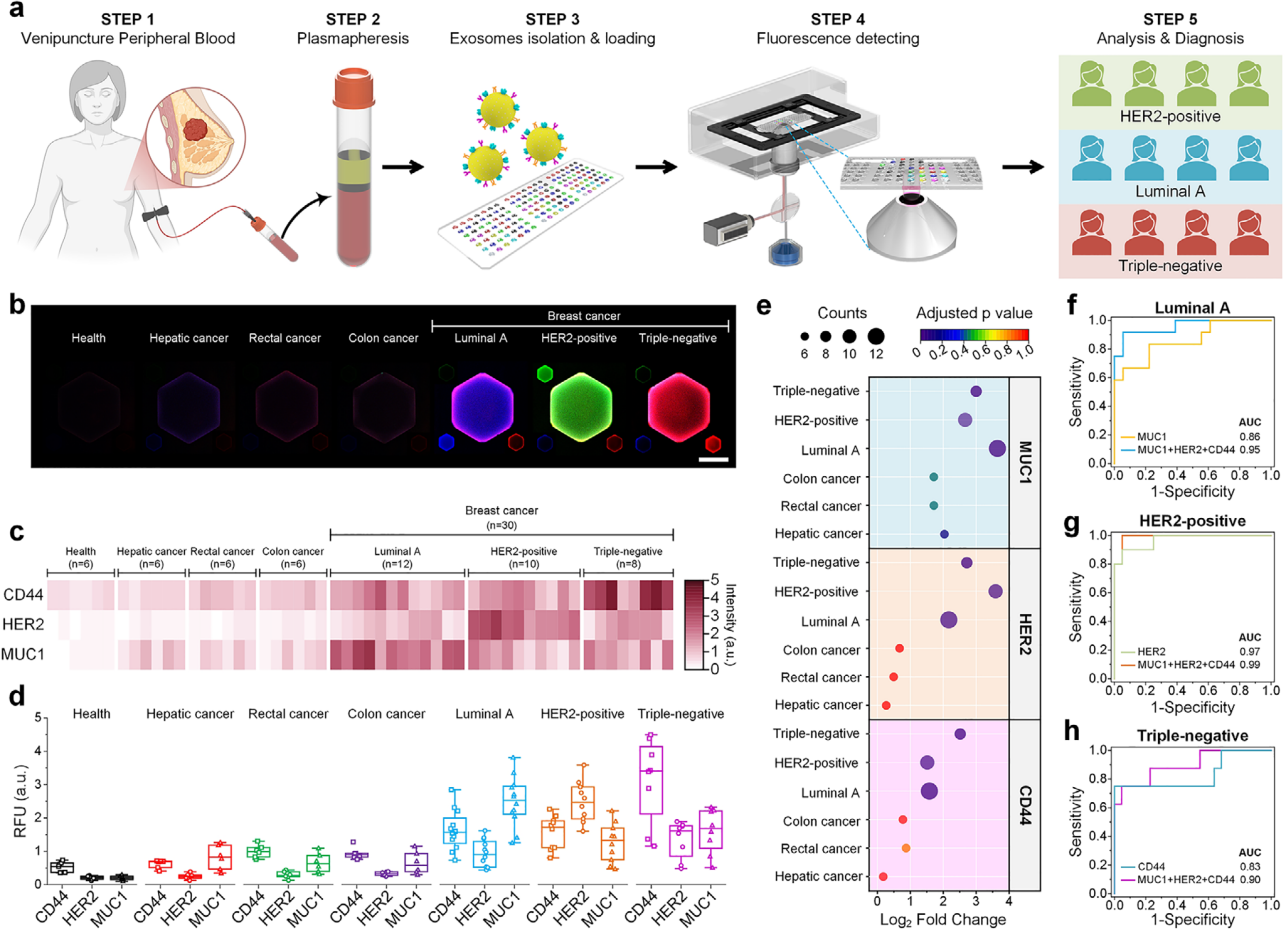
(a) Schematic illustration of the workflow for clinical plasma exosome detection. *Note*: Partial contents are adopted from BioRender. (b) Representative fluorescence images of the droplet arrays for the healthy group, hepatic cancer patients, rectal cancer patients, colon cancer patients, Luminal A patients, HER‐positive patients, and triple‐negative patients, respectively. Scale bar for merged images: 500 µm; the side length of the hexagonal pattern in each individual fluorescence channel is 600 µm. (c) Heatmap of fluorescence intensity of HER2‐aptamer‐FAM/MoS_2_ based sensing platform, MUC1‐aptamer‐Cy3/MoS_2_ based sensing platform, and CD44‐aptamer‐Cy5/MoS_2_ based sensing platform for detecting samples from healthy group (*n* = 6), hepatic cancer patients (n = 6), rectal cancer patients (*n* = 6), colon cancer patients (*n* = 6), and breast cancer patients (*n* = 30). (d) Fluorescence signals of HER2‐aptamer‐FAM/MoS_2_ based sensing platform, MUC1‐aptamer‐Cy3/MoS_2_ based sensing platform, and CD44‐aptamer‐Cy5/MoS_2_ based sensing platform in different groups. Box plot showing the median and the 25‐75 percentiles. (*n* = 6 for Health group, *n* = 6 for Hepatic cancer group, *n* = 6 for Rectal cancer group, *n* = 6 for Colon cancer group, *n* = 12 for Breast cancer Luminal A group, *n* = 10 for Breast cancer HER2‐positive group, *n* = 8 for Breast cancer Triple‐negative group). (e) Bubble plots showing the combined log_2_ fold changes and adjusted *p*‐values of the fluorescence signals of clinical samples detected by the aptasensor. ROC curves of single aptamer and multiplex aptamers for identifying (f) Luminal A patients, (g) HER‐positive patients, and (h) Triple‐negative patients. *Note*: Partial contents are created in BioRender. Liu, Y. (2026) https://BioRender.com/bl6hynm.

To develop the clinical potential of the MoS_2_ aptasensor for screening breast cancer subtypes, we tested exosomes isolated from the plasma of clinical samples. In this trial, the cohort included 6 healthy controls, 6 hepatic cancer patients, 6 rectal cancer patients, 6 colon cancer patients, and 30 breast cancer patients. The breast cancer cases were histologically classified into subtypes: 12 Luminal A, 10 HER2‐positive, and 8 Triple‐negative. The detection workflow is illustrated in Figure 4a. Briefly, peripheral blood was collected from all participants, followed by plasma isolation via centrifugation and subsequent exosome extraction by our customized method.

Prior to clinical detection, high sample purity was crucial, as plasma‐derived exosome preparations often contain contaminants that can compromise assay performance. To improve purity, we compared three isolation strategies: (1) PEG‐based precipitation using a commercial kit, (2) conventional ultracentrifugation with plasma diluted in PBS to meet the loading volume, and (3) our customized approach that combined kit‐based enrichment with a subsequent ultracentrifugation wash to remove nonspecific co‐precipitates, soluble plasma proteins, and free endogenous nucleases. Exosomes obtained by the three methods were then characterized by nanoparticle tracking analysis (NTA) (Figure S9a–d) and BCA‐based protein quantification (Figure S9e). The particle‐to‐protein ratio indicated that the customized protocol provided the highest purity among the three approaches (Figure S9f).

To further exclude false‐positive fluorescence recovery arising from endogenous nuclease activity in the purified exosome fraction, we compared the fluorescence signals of the aptamer sensors incubated with various purified exosome solutions in the presence or absence of EDTA, an endogenous nuclease inhibitor, without adding exogenous DNase I (Figure S10). For target‐specific exosome groups, EDTA(+) and EDTA(–) conditions showed no obvious difference in fluorescence recovery, whereas for non‐specific exosome groups, both conditions produced negligible fluorescence changes. These results indicate that endogenous nucleases in the isolated plasma exosome samples do not measurably contribute to signal generation, and that fluorescence recovery in our assay predominantly arises from specific exosome–aptamer interactions and the added exogenous DNase I.

Exosomes isolated from clinical plasma samples using the customized protocol were then added to droplet microarray chips pre‐coated with mixed HER2‐, MUC1‐, and CD44‐aptamer/MoS_2_ hybrids for multiplexed detection. After incubation for 1 h at room temperature in a humidified chamber, the chips were imaged by a laser scanning confocal microscope, and the representative fluorescence images are shown in Figure 4b. Fluorescence recovery was used as an indicator of the corresponding exosomal protein expression. The heatmap in Figure 4c summarizes the relative fluorescence intensities of all clinical samples. Each column represents one participant. Notably, the clinically defined breast cancer subtype did not always show the highest signal in its corresponding single‐marker row, indicating that subtype identification based on a single exosomal protein may be insufficient. The grouped fluorescence results (Figure 4d) further showed that MUC1, HER2, and CD44 signals were generally higher in breast cancer samples than in healthy controls and other cancer groups. Among the breast cancer subtypes, Luminal A samples showed the highest MUC1 signal, HER2‐positive samples showed the highest HER2 signal, and triple‐negative samples showed the highest CD44 signal, consistent with their expected molecular characteristics. To further assess group differences, log2 fold changes in fluorescence intensity relative to the healthy group were summarized in the bubble plot (Figure 4e), with bubble size representing sample number and color indicating the adjusted *p* value. Compared with healthy controls and other cancer groups, breast cancer subtypes showed larger fold changes and higher statistical significance for their corresponding aptamer signals. ROC analysis was then performed to evaluate the diagnostic performance of single‐marker and multiplex profiling strategies among the breast cancer cohort (Figure 4f–h). Because single‐protein analysis may increase subtype mismatch, we compared the performance of individual aptamers with that of the combined HER2/MUC1/CD44 signature. The multiplex strategy consistently achieved higher AUC values across all three subtypes. For Luminal A samples, the AUC increased from 0.86 (95% CI: 0.73–0.99) for MUC1 alone to 0.95 (95% CI: 0.89–1.00) for the multiplex profile. For HER2‐positive samples, the AUC increased from 0.97 (95% CI: 0.92–1.00) for HER2 alone to 0.99 (95% CI: 0.96–1.00) for the multiplex profile. For triple‐negative samples, the AUC increased from 0.83 (95% CI: 0.69–0.98) for CD44 alone to 0.90 (95% CI: 0.78–1.00) for the multiplex profile. These results demonstrate that multiplex exosomal protein profiling improves subtype discrimination and reduces potential false‐positive classification caused by reliance on a single marker. Together, these biomarkers were not intended to serve as absolutely subtype‐specific markers individually; rather, they were incorporated as components of a combinatorial exosomal signature for subtype discrimination. Overall, the MoS_2_ aptasensor showed reliable and sensitive performance in clinical plasma samples, supporting its potential as a practical tool for breast cancer subtyping in clinical diagnostics.

Although our aptasensor performed robustly in multiplex detection of cell line‐derived exosomes, plasma‐derived exosomes are inherently heterogeneous and include vesicles released from immune cells and other non‐tumor sources. Western blot analysis (Figure S11) showed that peripheral blood mononuclear cells (PBMC)‐derived exosomes and healthy donor plasma exosomes exhibited visible but very faint bands for HER2, MUC1, and CD44, consistent with low basal expression rather than overexpression, whereas CD63 remained strongly expressed in both groups. These results indicate that non‐tumor‐derived exosomes may share certain target proteins with tumor‐derived exosomes but do not display the characteristic overexpression patterns of breast cancer subtypes. Importantly, our subtype identification strategy relies on the combined expression profile of HER2, MUC1, and CD44 rather than any single marker, thereby minimizing interference from background exosomes in plasma samples.

### 2.6 Single‐Blind Validation of the Assay in Clinical Samples

**FIGURE 5:**
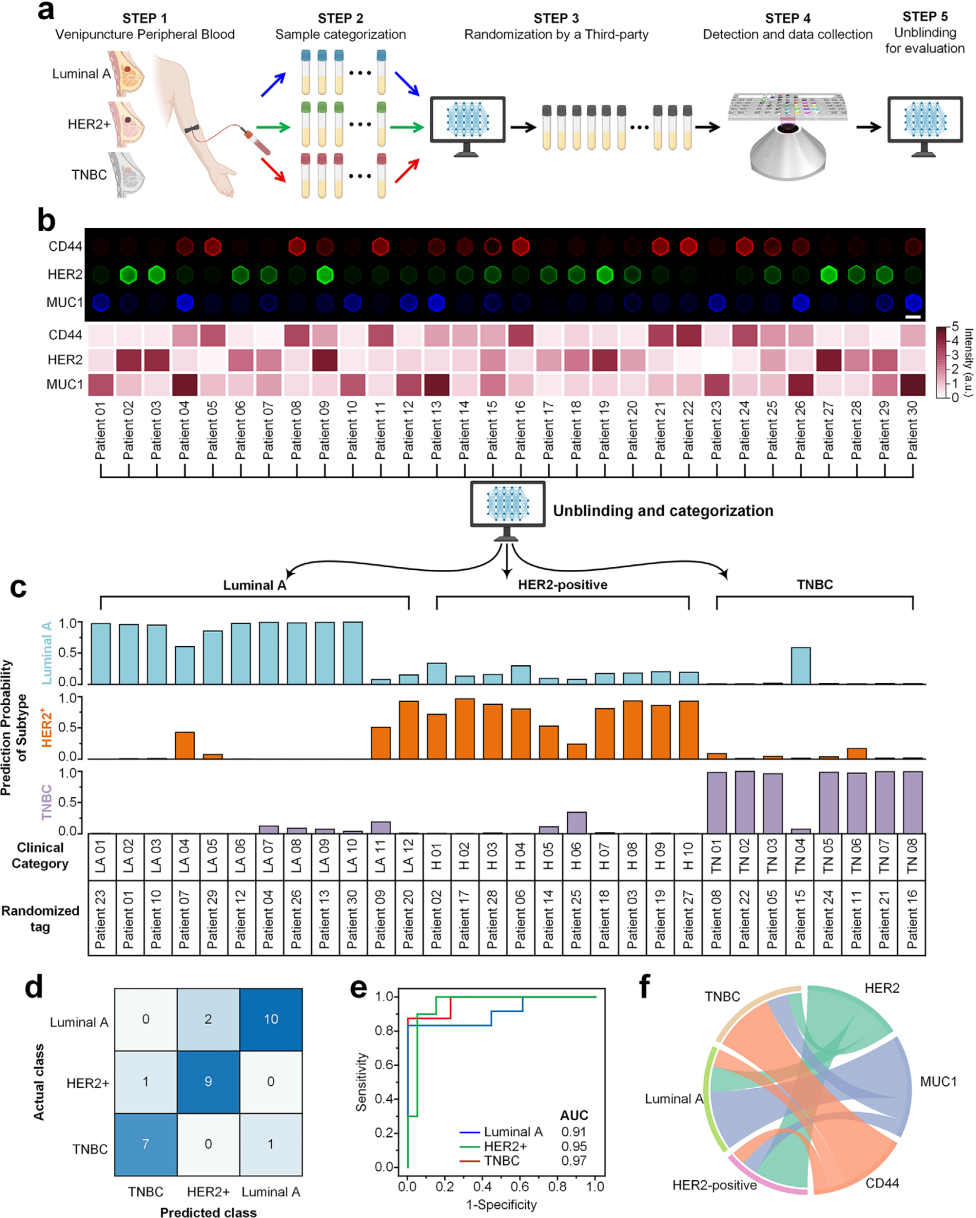
(a) Schematic illustration of the workflow for single‐blind detection of breast cancer subtypes in a breast cancer cohort. *Note*: Partial contents are adopted from BioRender. (b) Assembled fluorescence images of the droplet array for randomly tagged patient samples and the corresponding summarized heatmap. Scale bar: 1 mm. (c) Calculated prediction probabilities for Luminal A, HER2‐positive, and triple‐negative breast cancer subtypes, respectively. (d) Confusion matrix summarizing the prediction performance of multiple exosomal biomarkers for breast cancer subtype identification. (e) ROC curves of the multiplex aptamers for identifying Luminal A patients, HER2‐positive patients, and Triple‐negative patients in the single‐blind validation trial. (f) Chord diagram showing the relative fluorescence recovery ratios of the aptasensor in response to the three breast cancer subtype cohorts. *Note*: Partial contents are created in BioRender. Liu, Y. (2026) https://BioRender.com/xztoayv.

To further evaluate the diagnostic performance of the developed droplet microarray chip‐based aptasensor, we conducted a single‐blind validation using an independent cohort of breast cancer plasma samples. As illustrated in Figure 5a, plasma samples from breast cancer patients were first categorized according to subtype information obtained from IHC reports and diagnostic records, after which a third party then assigned random codes to all samples. Exosomes were then isolated using the customized protocol and subsequently detected on the droplet microarray chip. After all fluorescence images were acquired, the sample identities were unblinded for further data analysis.

Representative assembled fluorescence images and corresponding heatmaps for each channel are shown in Figure 5b, arranged by randomized sample codes. Using the relative fluorescence recovery ratios, we calculated the predicted subtype probability for each participant. After unblinding by the third party, the samples were regrouped according to their confirmed subtypes (Figure 5c), and the prediction results were compared with the true labels in a confusion matrix (Figure 5d), yielding an overall subtype classification accuracy of 86.7%. ROC curve analysis was then conducted to evaluate the multiplex profiling performance in this single‐blind trial (Figure 5e). The AUC values were 0.91 (95% CI: 0.81–1.00) for Luminal A, 0.95 (95% CI: 0.85–1.00) for HER2‐positive, and 0.97 (95% CI: 0.91–1.00) for triple‐negative subtypes. The differences in AUC values between the two clinical validation cohorts may reflect inter‐cohort heterogeneity, such as variation in patient characteristics and exosome abundance, as well as the limited sample size in each cohort. Nonetheless, both cohorts demonstrated consistently high diagnostic performance, supporting the robustness and clinical applicability of the proposed aptasensor. Finally, a chord diagram (Figure 5f) was used to visualize the relative fluorescence recovery ratios across subtypes, further highlighting the characteristic biomarker expression patterns.

## 3 Discussion and Conclusion

In this study, we developed and validated a droplet microarray platform integrated with a DNase I‐assisted MoS_2_‐based FRET aptasensor for the multiplexed detection and profiling of breast cancer exosome subtypes. Our results demonstrate that this platform enables highly sensitive and specific identification of exosomal protein biomarkers—HER2, MUC1, and CD44—allowing for accurate discrimination among luminal A, HER2‐positive, and triple‐negative breast cancer subtypes. The incorporation of DNase I‐mediated signal amplification significantly enhanced detection sensitivity, achieving a remarkable limit of detection of approximately 10^2^ particles/mL. Multiplexed fluorescence analysis on the droplet microarray chip provided clear and distinct fluorescence patterns corresponding to different exosome subtypes, and the RGB color model facilitated straightforward visual identification of single and mixed exosome populations. Clinical validation using plasma‐derived exosomes from a diverse patient cohort further confirmed the platform's robust performance, with multiplex protein profiling yielding higher predictive accuracy (AUC values up to 0.99) compared to single biomarker approaches. Importantly, this method demonstrated rapid turnaround, minimal sample consumption, and compatibility with high‐throughput analysis, addressing key limitations of conventional exosome‐based diagnostics. Collectively, our findings highlight the potential of this droplet microarray multiplexed FRET aptasensor as a powerful tool for precise breast cancer subtype classification, which could significantly enhance diagnostic accuracy and inform personalized treatment strategies in clinical oncology. This advancement represents a promising step forward in the application of exosome‐based liquid biopsy for precision medicine.

## 4 Experimental Section

### 4.1 Materials

MoS_2_ nanosheets were purchased from XFNANO (Nanjing, China). DNase I, Amplification Grade (cat. 18068015), DNase/RNase‐Free Distilled Water (cat. 10977023), Total Exosome Isolation Reagent (from plasma), and the Micro BCA Protein Assay Kit were obtained from Thermofisher Scientific (MA, US). Synthesized HER2 DNA‐aptamer, MUC1‐aptamer, and CD44 aptamer were purchased from Sangon Biotech (Shanghai, China). The following sequences were adopted:

HER2 Aptamer: 5′‐FAM‐GGG CCG TCG AAC ACG AGC ATG GTG CGT GGA CCT AGG ATG ACC TGA GTA CTG TCC‐3′ [48]

MUC1 Aptamer: 5′‐Cy3‐GCA GTT GAT CCT TTG GAT ACC CTG G‐3′ [49]

CD44 aptamer: 5′‐Cy5‐ GAG ATT CAT CAC GCG CAT AGT CCC AAG GCC TGC AAG GGA ACC AAG GAC ACA GCG ACT ATG CGA TGA TGT CTT C‐3′ [50]

### 4.2 Cell Culture

MDA‐MB‐231 and SKBR3 cells were cultured in high‐glucose Dulbecco's Modified Eagle's Medium (DMEM; Invitrogen) supplemented with 10% fetal bovine serum (FBS; Gibco, Brazil) and 1% penicillin‐streptomycin (10,000 U/mL). MCF‐7 cells were maintained in RPMI 1640 medium (Invitrogen) supplemented with 10% FBS and 1% penicillin‐streptomycin. MCF‐10A cells were grown in mammary epithelial basal medium (MEBM; CC‐3151, Lonza) supplemented with the provided supplements pack (CC‐4136, Lonza), which contains bovine pituitary extract (BPE), human epidermal growth factor (hEGF), insulin, hydrocortisone, and gentamicin‐amphotericin (GA‐1000). All cell lines were incubated at 37°C in a humidified atmosphere containing 5% CO_2_. The culture medium was replaced every two days. Upon reaching 75% confluency, cells were detached using 0.25% trypsin–EDTA in PBS, and subsequently subcultured or harvested for experiments. Cells were used between passages 3 and 12.

### 4.3 Characterization of MoS_2_ and DNA Aptamer

Transmission electron microscopy (TEM, JEOL‐2100F) was used to characterize the lateral size of the MoS_2_ nanosheets. UV–vis spectrophotometer (Ultrospec 2100 pro) was utilized to characterize the MoS_2_ absorbance spectrum. Zetasizer Nano Z system (Malvern Instruments Ltd) was used to measure the MoS_2_ size distribution and quantify the zeta potential. Excitation and emission spectrums of DNA aptamer were recorded by photoluminescence spectrometer (Edinburgh FS5). The surface profile of micropatterned MoS_2_ on the droplet array chip was examined by the Atomic Force Microscopy (MultiMode 8, Bruker).

### 4.4 Construction of Aptamer‐MoS_2_ Based FRET Biosensor

MoS_2_ nanosheets were sonicated for a sufficient time, and then double‐distilled water was added, followed by centrifuge to achieve a homogeneous aqueous solution. MoS_2_ aqueous solutions with final concentrations ranging from 0 to 200 µg/mL were mixed with FAM‐conjugated HER2‐aptamer (40 nm), Cy3‐conjugated MUC1‐aptamer (40 nm), and Cy5‐conjugated CD44‐aptamer (40 nM) in DNase/RNase‐Free Distilled Water for 5 min to quench the fluorescence of the corresponding aptamers. After that, the fluorescent signal of all the samples was measured by a laser scanning confocal microscope (SP8, Leica Microsystems). Besides, the size and zeta potential of MoS_2_/Aptamer conjugates were measured by Zetasizer Nano Z system.

### 4.5 Isolation of Exosomes From Cell Medium

Exosomes are extracted from cell culture medium by using an ultracentrifuge (Optima XPN‐100, Beckman). Before isolating exosomes, MCF‐7 cells, SKBR3, and MDA‐MB‐231 cells were cultured to achieve a high cell density (1‐2 million/mL). Then, the completed medium was replaced by a conditional medium containing exosome‐depleted FBS and cultured for an additional 2 days. After that, the cell culture medium was collected and centrifugated at 300 × g for 10 mins to preliminarily remove debris and dead cells. Next, the cell culture medium was first centrifuged at 2,000 × g for 10 mins to exclude residual debris, and then at 10,000 × g for 30 mins to further remove dead cells, debris, and other undesired large particles such as shedding vesicles and apoptotic bodies. Then the cell culture medium was centrifuged at 100,000 × g for 90 mins. After that, the supernatant was discarded, and the pellet was resuspended in PBS followed by centrifuge at 100,000 × g for another 90 mins. Finally, the exosomes can be extracted in a pellet and resuspended in PBS. All centrifugations were operated at 4°C.

### 4.6 Isolation of Exosomes From Clinical Human Plasma

This study was approved by the Ethics Committee of Guangdong Provincial People's Hospital (Guangdong Academy of Medical Sciences), Southern Medical University, Guangzhou, China (Ethics No. KY2025‐037‐01). Written informed consent was obtained from all patients and healthy participants prior to enrollment, and the consent procedure was also approved by the ethics committee. In the first trial, a total of 54 clinical plasma samples were collected from the Department of Laboratory Medicine, Guangdong Provincial People's Hospital, Guangzhou, China. Basic demographic information, including sex and age, for the healthy group (*n* = 6), hepatic cancer patients (*n* = 6), rectal cancer patients (*n* = 6), colon cancer patients (*n* = 6), and breast cancer patients (*n* = 30), is summarized in Table S1. Partial immunohistochemical results from breast cancer tissue samples, including ER, PR, HER2, and Ki‐67, are also provided in Table S1 for breast cancer subtype classification.

In the second single‐blind trial, a total of 30 plasma samples from breast cancer patients were also obtained from the Department of Laboratory Medicine, Guangdong Provincial People's Hospital. These samples were randomly coded by a third party prior to analysis. After detection using the aptasensor developed in this study, the sample identities were unblinded and categorized by the same third party. The corresponding patient information was then disclosed, and their basic demographic information, together with partial immunohistochemical results, including ER, PR, HER2, and Ki‐67, is provided in Table S2.

Approximately 1 mL of plasma was collected from the peripheral blood of each participant. Owing to the limited plasma volume available for direct ultracentrifugation, a customized exosome isolation procedure combining a commercial isolation reagent with ultracentrifugation was employed. Briefly, plasma exosomes were first pre‐enriched using the Total Exosome Isolation Reagent (from plasma) according to the manufacturer's instructions. The resulting exosome pellet was resuspended in PBS and transferred to a sterilized 13.2 mL thin‐wall ultracentrifuge tube (Beckman). The sample was then subjected to ultracentrifugation (Optima XPN‐100, Beckman) at 100,000 × g and 4°C for 90 min to improve sample purity. After the ultracentrifugation, the purified exosomes were resuspended in 50 µL PBS and stored at −20°C until further use.

### 4.7 Characterization of Exosomes

The morphology of exosomes was characterized by Transmission electron microscopy (TEM). First, the carbon‐coated copper grids were treated by the PELCO easiGlow Glow Discharge Cleaning System to make the surface negatively charged and ensure that the carbon film surface is hydrophilic. Then the exosomes were pipetted on the hydrophilic treated carbon‐coated copper grid for absorption for 30 s. Next, the copper grid was negatively stained with uranyl acetate for 45 s. Lastly, the grids were dried at room temperature and imaged by Talos L120C TEM (ThermoFisher, US). The size and concentrations of exosomes were characterized by Nanoparticle Tracking Analysis (NanoSight NS300, Malvern Panalytical). Briefly, the exosomes were diluted in 1 mL PBS. After loading via a syringe pump, the particles were illuminated with a 405 nm laser beam and captured by a digital camera.

### 4.8 Western Blotting Analysis

Western blotting was performed to identify common exosome markers (CD63) and specific breast cancer exosome markers (HER2, MUC1, and CD44). Protein concentrations were determined using a BCA assay kit, and samples were diluted with PBS to a final concentration of 100 ng/mL for electrophoresis. The diluted proteins were mixed with 4× Laemmli buffer and heated at 95°C for 10 min to denature. Proteins were separated by SDS‐PAGE and transferred onto 0.45 µm nitrocellulose membranes at 150 mA for 30 min. Membranes were blocked in TBS‐T containing 5% non‐fat milk for 1 h at room temperature, followed by overnight incubation with primary antibodies against HER2, MUC1, CD44, and CD63. After washing, membranes were incubated with the corresponding HRP‐conjugated secondary antibodies for 1 h at room temperature. Immunoreactive bands were visualized using enhanced chemiluminescence (ChemiDoc Imaging System, Bio‐Rad).

### 4.9 Agarose Gel Electrophoresis of Aptamers

To prepare the agarose gel, 4 g of agarose was dissolved in 100 mL of 0.5× TBE buffer by heating in a microwave until fully dissolved. After cooling to approximately 60°C, 10 µL of SYBR Gold Nucleic Acid Gel Stain was added to the solution with thorough swirling. The mixture was then poured into a gel tray and allowed to solidify at room temperature. For the assay, 40 nm of HER2‐aptamer‐FAM, MUC1‐aptamer‐Cy3, and CD44‐aptamer‐Cy5 were incubated with or without DNase I enzyme (1 U) and MoS_2_, as appropriate. Samples were then mixed with gel loading buffer and loaded into the wells of the prepared agarose gel. Electrophoresis was carried out in 0.5× TBE buffer at 80 V for approximately 1.5 h. DNA bands labeled with SYBR Gold were visualized using enhanced chemiluminescence.

### 4.10 Fabrication of Droplet Array Chip

The droplet array chip is fabricated by hydrophobic treating of micropattern‐covered chips (Figure S2). Briefly, glass slides (Citoglas, Jiangsu) were rinsed with acetone, isopropyl alcohol (IPA), and DI water, respectively, to clean the surface. The cleaned glass slides were treated by oxygen plasma cleaner (PDC‐002‐HP, Harrick Plasma) to further clean and activate the surface. Then glass slides were spin‐coated with AZ5214‐E (MicroChem) positive resist at 3000 rpm for 30s, and then soft‐baked on a hotplate at 110°C for 5 min. After cooling down to room temperature, slides were exposed under a mask aligner (SUSS MA6, SUSS MicroTec) for 13.5s with the energy density at 9.8 mW/cm^2^. The exposed slides were developed in AZ300 MIF (MicroChem) and rinsed with DI water, then dried with nitrogen gas and baked on a hotplate at 110°C for an extra 5 min. Before placing the developed glass slides in a vacuum desiccator for silanization (Trichloro(1H,1H,2H,2H‐perfluorooctyl) silane, Sigma Aldrich) overnight, the developed slides were treated by oxygen plasma cleaner for another 1 min to facilitate the adsorption of silane. The silane slides were then immersed in acetone to lift off the protective AZ5214E pattern layer, followed by rinsing with IPA, DI water, and drying with nitrogen gas.

### 4.11 Droplet Microarray Chip for Multiplex Detection of Three Subtypes of Breast Cancer Exosomes

For multiplex exosome detection, a series of MoS_2_ concentrations (0, 25, 50, 100, 150, and 200 µg/mL) was applied to the hydrophilic spots on the chip by continuous wetting. After drying at room temperature, 40 nM FAM‐HER2 aptamer, Cy3‐MUC1 aptamer, and Cy5‐CD44 aptamer were added to the MoS_2_‐coated spots. The chip was then placed in a 100% humidified chamber and incubated for 5 min. Confocal imaging was performed to analyze the quenching effect. For the detection process, 200 µg/mL MoS_2_ was applied to cover all hydrophilic spots in the corresponding columns of the chip and dried at room temperature. Aptamer combinations were then added to the corresponding rows, followed by incubation in a 100% humidified chamber for 5 min. Subsequently, different combinations of exosomes were added to the corresponding rows by continuous wetting, and the chip was incubated in a 100% humidified chamber for 1 h. Fluorescence images were captured using confocal microscopy (SP8, Leica Microsystems), and fluorescence intensity was quantified using ImageJ software (NIH). All experiments were performed in triplicate.

### 4.12 Microscope Settings for Multiplex Detection

Fluorescence images for multiplex exosome detection were acquired using a laser scanning confocal microscope (SP8, Leica Microsystems) with optimized multichannel acquisition settings. Sequential scanning in the “between frames” mode was employed, in which the same region was scanned sequentially by three laser lines to minimize spectral crosstalk. Three hybrid detectors (HyD) were used for fluorescence signal collection. The emission detection windows were optimized using Leica Dye Assistant to maximize coverage of the fluorescence emission peaks while avoiding spectral overlap. Specifically, HER2 aptamer‐FAM was imaged using 488 nm excitation, and the emission signal was collected with HyD 1 over the range of 493–547 nm. MUC1 aptamer‐Cy3 was imaged using 552 nm excitation, and the emission signal was collected with HyD 2 over the range of 557–642 nm. CD44 aptamer‐Cy5 was imaged using 638 nm excitation, and the emission signal was collected with HyD 3 over the range of 643–778 nm. Before each imaging experiment, equal concentrations of dye‐labeled aptamers were loaded onto the droplet sites, and the acquisition parameters were empirically fine‐tuned to ensure balanced fluorescence signals across all channels and clear visualization of the merged hexagonal pattern in white.

### 4.13 Statistical Analysis

Statistical analyses were performed using GraphPad Prism (GraphPad Software, MA, USA). Statistical significance was determined using the unpaired Student's t‐test. No data transformation or outlier exclusion was applied prior to statistical testing. Error bars in the figures represent standard errors or standard deviations, as detailed in the respective figure descriptions. Sample numbers (n) are indicated in the respective figure captions. Patients’ numbers are provided in the main text, and detailed group information and corresponding numbers are provided in the Supporting Information. *P* values less than 0.05 were considered statistically significant and denoted as ^*^ *p* < 0.05.

## Author Contributions

Y.L., R.Z., and M.Y. conceived the study and designed experiments. M.Y., B.G., and Y.T. supervised the study. Y.L and R.Z performed the experiments and analyzed the experimental data. Y.L and J.S (Jialei Song) designed and fabricated the droplet microarray chip. M.X. and Y.M. collected the clinical samples and summarized patients’ information. Y.L., R.Z., J.S. (Jingyu Shi), and M.Y wrote the manuscript. Z.L. provided comments on the manuscript. All authors helped with the interpretation of the results and approved the manuscript.

## Conflicts of Interest

The authors declare no conflicts of interest.

## Supporting information




**Supporting File**: smll74377‐sup‐0001‐SuppMat.pdf.

## Data Availability

The data that support the findings of this study are available in the supplementary material of this article.
