# Supplementary material for: A Droplet‐Microarray Platform for Multiplex Profiling of Breast Cancer Exosome Subtypes in Patients’ Blood Plasma Samples
Source: Small. 2026 Jul 6;22(49):e13591. doi: 10.1002/smll.202513591 (PMC13533275; doi:10.1002/smll.202513591)
Supplement: Supplementary file 1 [file SMLL-22-e13591-s001.pdf]

Supporting Information

A droplet-microarray platform for multiplex profiling of breast cancer exosome subtypes in patients' blood plasma samples

Yi Liu[1,2#], Ruolin Zhang[1,2#], Jialei Song[1], Meng Xiao[1,5], Yue Meng[5], Zhiyang Li[7], Jingyu Shi[1], Yunyan Tai[6,*], Bing Gu[5,*] and Mo Yang[1,2,3,4*]

[1]Department of Biomedical Engineering, The Hong Kong Polytechnic University, Hung Hom, Hong Kong SAR, China

[2]The Hong Kong Polytechnic University Shenzhen Research Institute, Shenzhen 518000, China

[3]Joint Research Center of Biosensing and Precision Theranostics, The Hong Kong Polytechnic University, Kowloon, Hong Kong 999077, China

[4]Research Centre for Nanoscience and Nanotechnology, The Hong Kong Polytechnic University, Kowloon, Hong Kong 999077, China

[5]Laboratory Medicine, Guangdong Provincial People's Hospital (Guangdong Academy of Medical Sciences), Southern Medical University, Guangzhou, Guangdong 510080, China

[6]Department of Oncology, Renmin Hospital, Hubei University of Medicine, Shiyan, Hubei 442000, P.R. China

[7]Department of Clinical Laboratory Medicine, Nanjing Drum Tower Hospital Clinical College of Jiangsu University, Zhongshan Road 321, Nanjing, 210008, Jiangsu Province, China

[*]Correspondence should be addressed to Yunyan Tai (email address: taiyunyan1@shiyanhospital.com), Bing Gu (email address: gubing@gdph.org.cn) and, Mo Yang (email address: mo.yang@polyu.edu.hk)

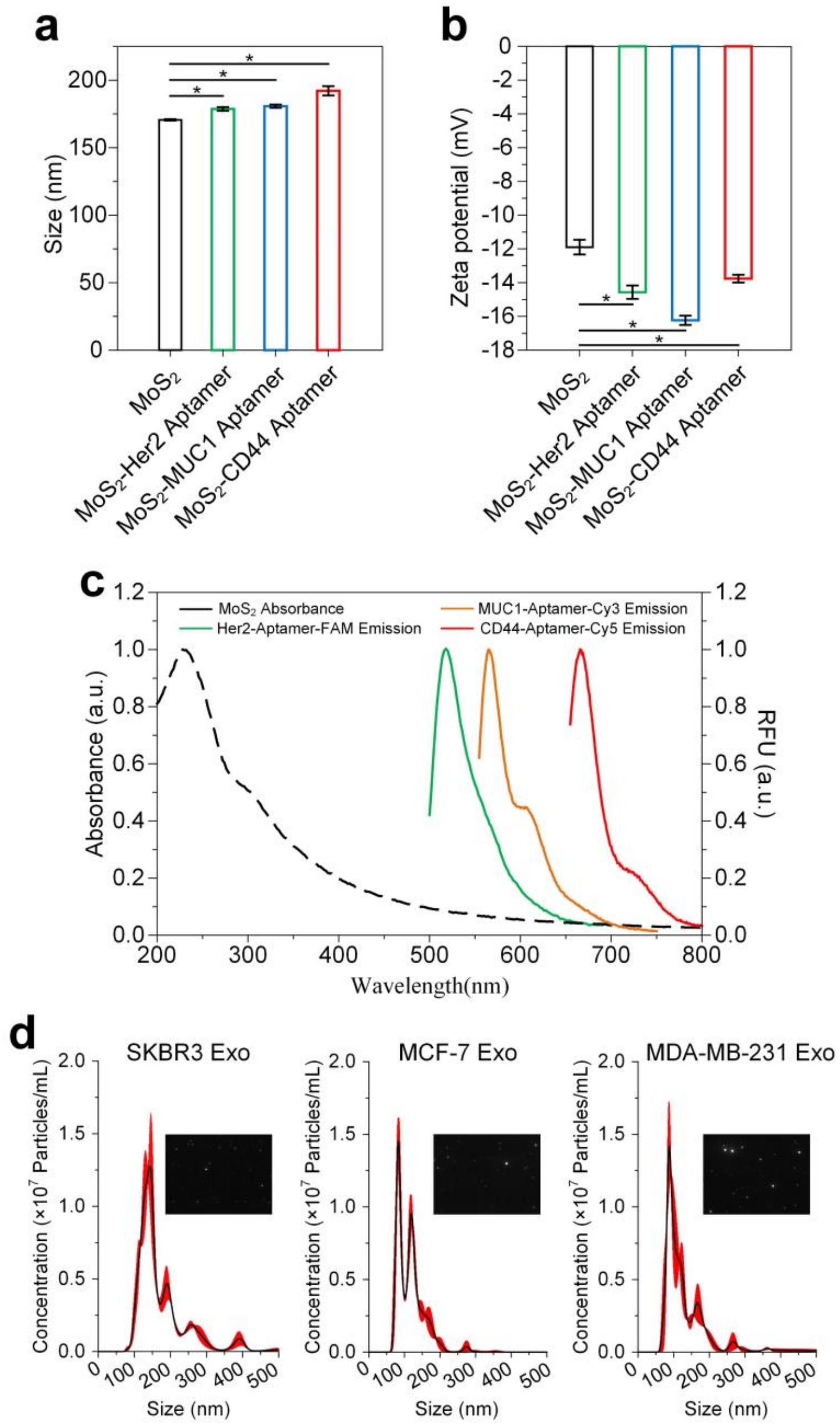

Fig. S1. (**a**) Hydrodynamic size and (**b**) zeta potential of MoS_2 nanosheets, the MoS_2-FAM-HER2 aptamer hybrid, the MoS_2-Cy3-MUC1 aptamer hybrid, and the MoS_2-Cy5-CD44 aptamer hybrid. (**c**) UV-Vis absorption spectrum of MoS_2 nanosheets and the fluorescence emission spectra of the aptamers. (**d**) Nanoparticle tracking analysis (NTA) of exosomes derived from SKBR3, MCF-7, and MDA-MB-231 cells. Data presented as mean ± SEM, $n = 3$. * presents the p-value < 0.05.

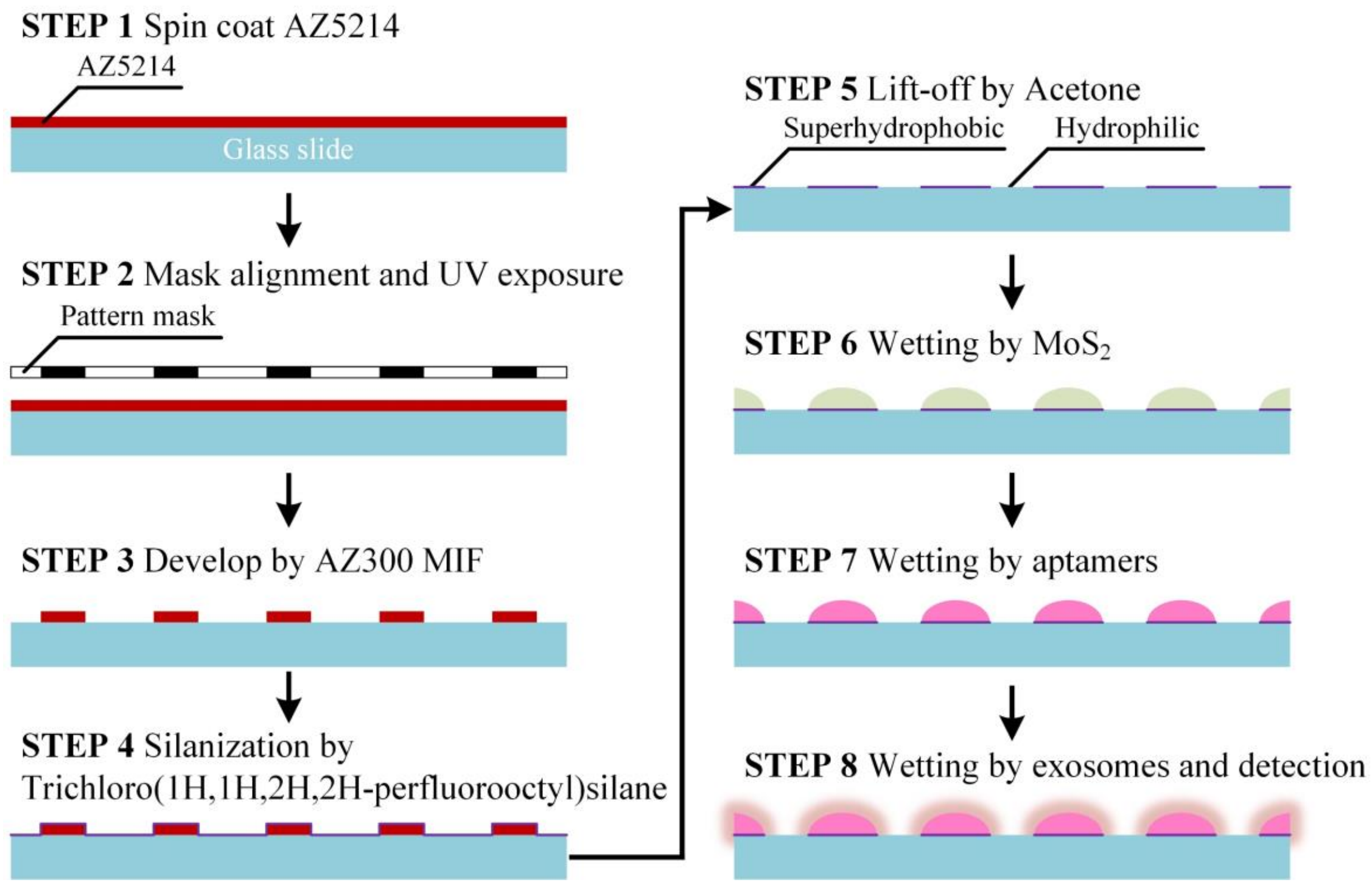

Fig. S2. Fabrication process of the droplet array chip for multiplex MoS_2-based aptasensing of breast cancer exosome subtypes.

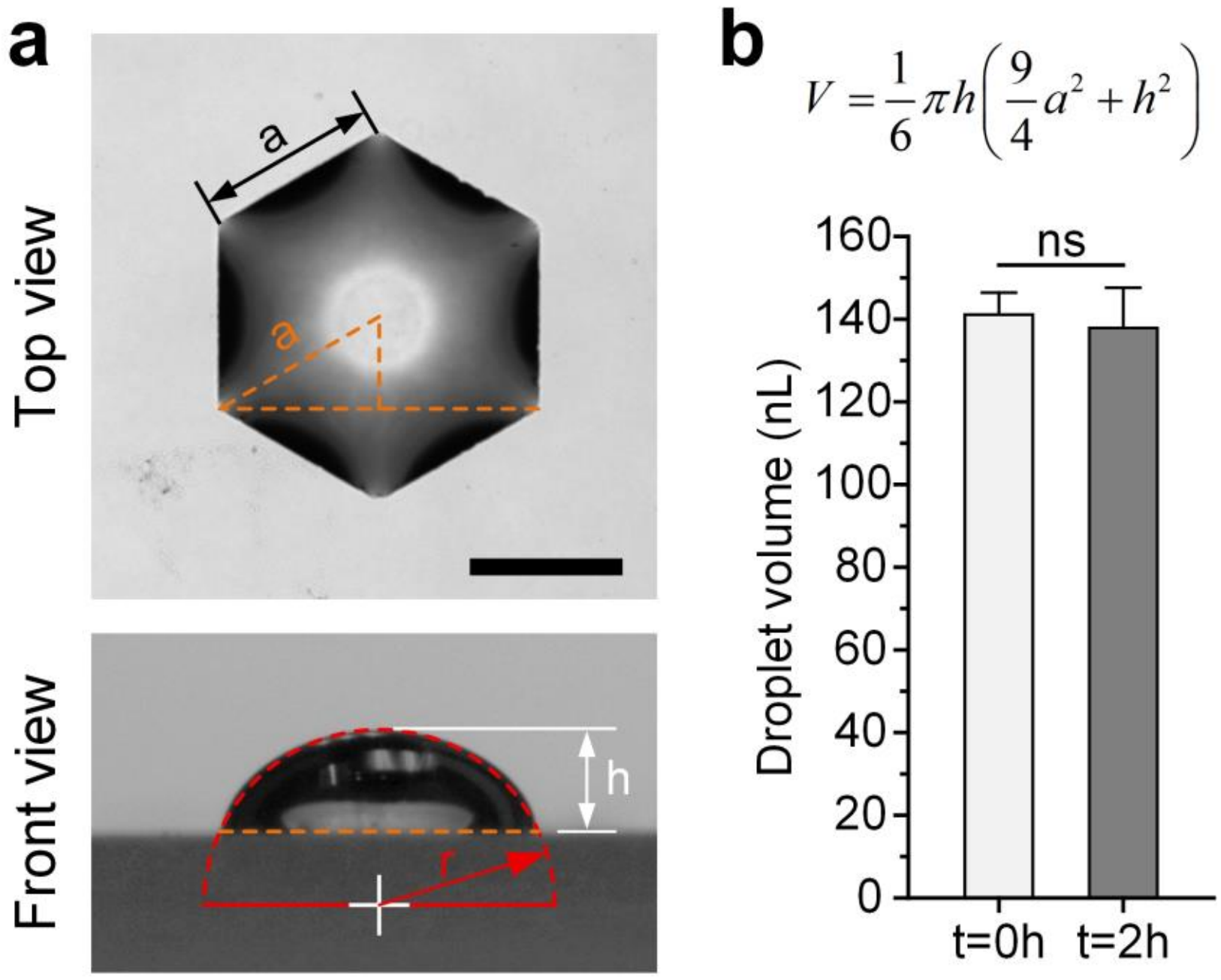

Fig. S3. (**a**) Geometric schematic of a single droplet on the droplet microarray chip. Scale bar: 500 μm. (**b**) Approximation formula used for droplet volume calculation and the corresponding droplet volumes at different time points, with the droplet maintained in a humidified chamber during the evaluation. Data presented as mean ± SEM, n = 3, ns presents not significant.

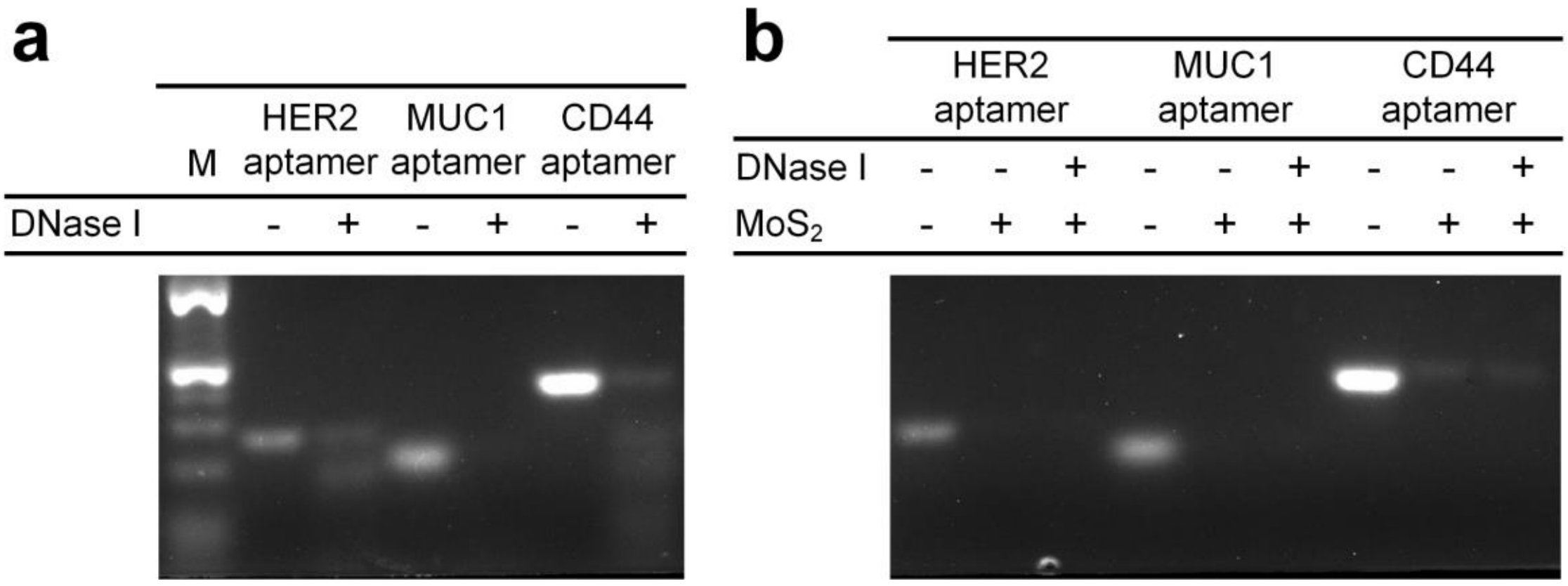

Fig. S4 . (**a**) Agarose gel electrophoresis images used to evaluate DNase I activity. (**b**) Agarose gel electrophoresis images demonstrating the fluorescence-quenching effect of MoS_2 and its protective effect against DNase I digestion.

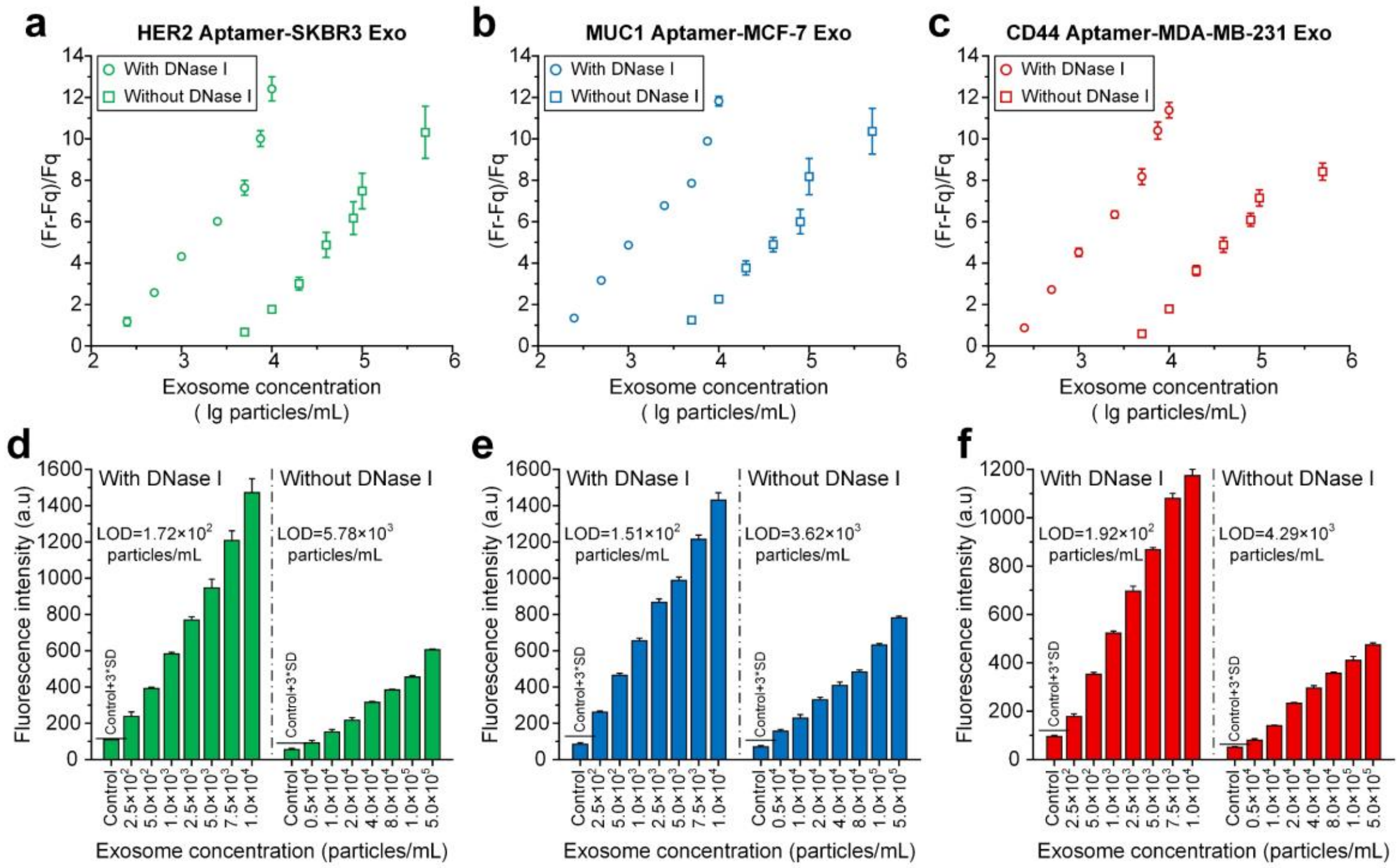

Fig. S5. (**a**) HER2 aptamer fluorescence recovery rate against logarithmic concentration of SKBR3 exosome in droplet sensing platform. (**b**) MUC1 aptamer fluorescence recovery rate against logarithmic concentration of MCF-7 exosome in droplet sensing platform. (**c**) CD44 aptamer fluorescence recovery rate against logarithmic concentration of MDA-MB-231 exosome in droplet sensing platform. (**d**) Quantitative analysis of fluorescence intensity of the droplet spot coated with MoS_2-HER2 aptamer hybrid doping a series of concentrations of SKBR3 exosomes with or without DNase I addition. (**e**) Quantitative analysis of fluorescence intensity of the droplet spot coated with MoS_2-MUC1 aptamer hybrid doping a series of concentrations of MCF-7 exosomes with or without DNase I addition. (**f**) Quantitative analysis of fluorescence intensity of the droplet spot coated with MoS_2-CD44 aptamer hybrid doping a series of concentrations of MDA-MB-231 exosomes with or without DNase I addition. Data presented as mean ± SEM, n = 3.

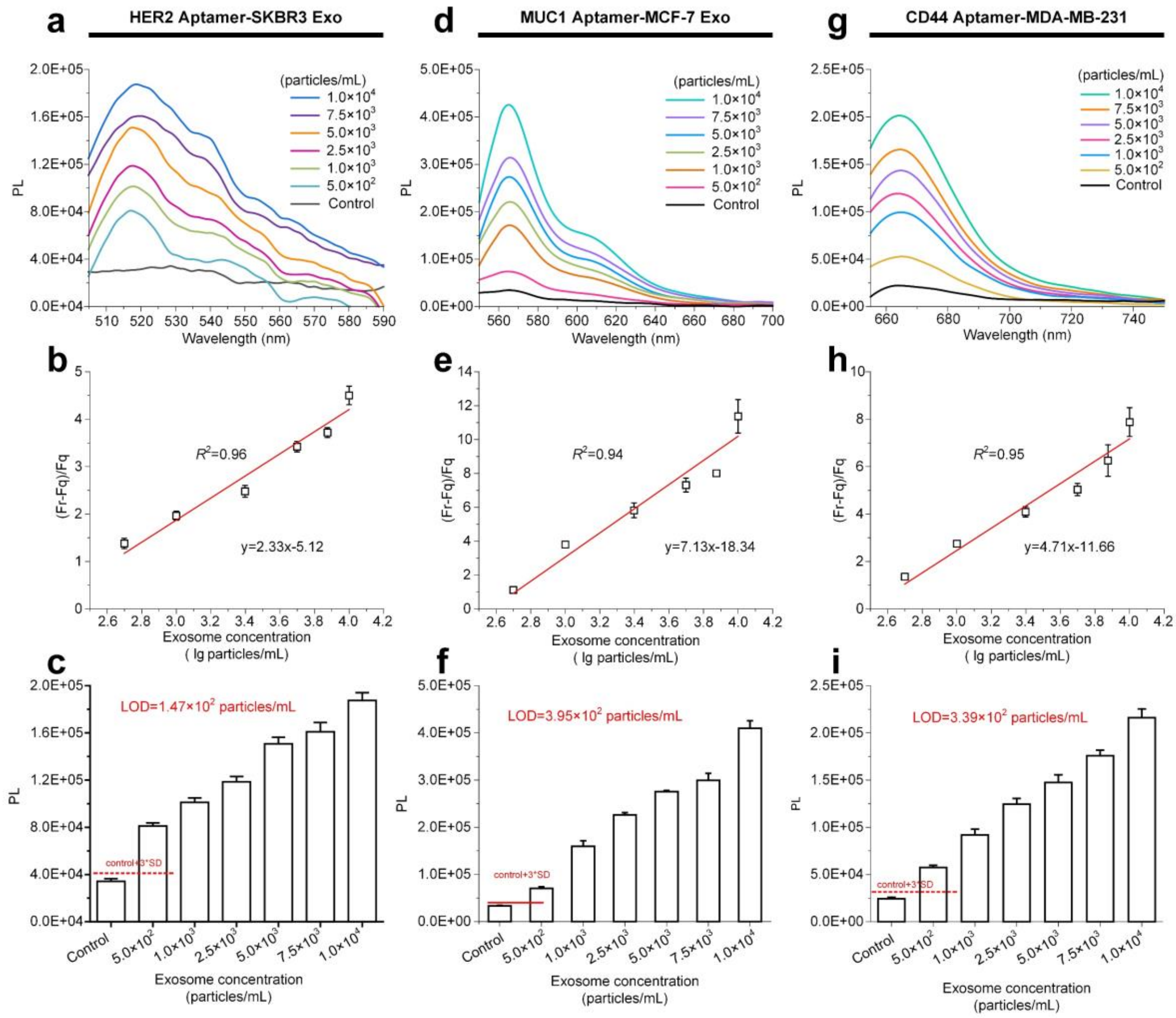

Fig. S6. (**a**) Fluorescence recovery spectra of the HER2-aptamer-FAM/MoS_2 sensing platform in response to SKBR3-derived exosomes at various concentrations. (**b**) Linear relationship between fluorescence recovery efficiency ((F_r-F_q)/F_q) and the logarithm of the concentrations of SKBR3-derived exosomes. (**c**) Plot of recovered peak fluorescence intensity versus SKBR3-derived exosome concentration for determination of the limit of detection (LOD). (**d**) Fluorescence recovery spectra of the MUC1-aptamer-Cy3/MoS_2 sensing platform in response to MCF-7-derived exosomes at various concentrations. (**e**) Linear relationship between fluorescence recovery efficiency ((F_r-F_q)/F_q) and the logarithm of the concentrations of MCF-7-derived exosomes. (**f**) Plot of recovered peak fluorescence intensity versus MCF-7-derived exosome concentration for determination of the limit of detection (LOD). (**g**) Fluorescence recovery spectra of the CD44-aptamer-Cy5/MoS_2 sensing platform in response to MDA-MB-231-derived exosomes at various concentrations. (**h**) Linear relationship between fluorescence recovery efficiency ((F_r-F_q)/F_q) and the logarithm of the

concentrations of MDA-MB-231-derived exosomes. (**i**) Plot of recovered peak fluorescence intensity versus MDA-MB-231-derived exosome concentration for determination of the limit of detection (LOD). Data presented as mean ± SEM, n = 3.

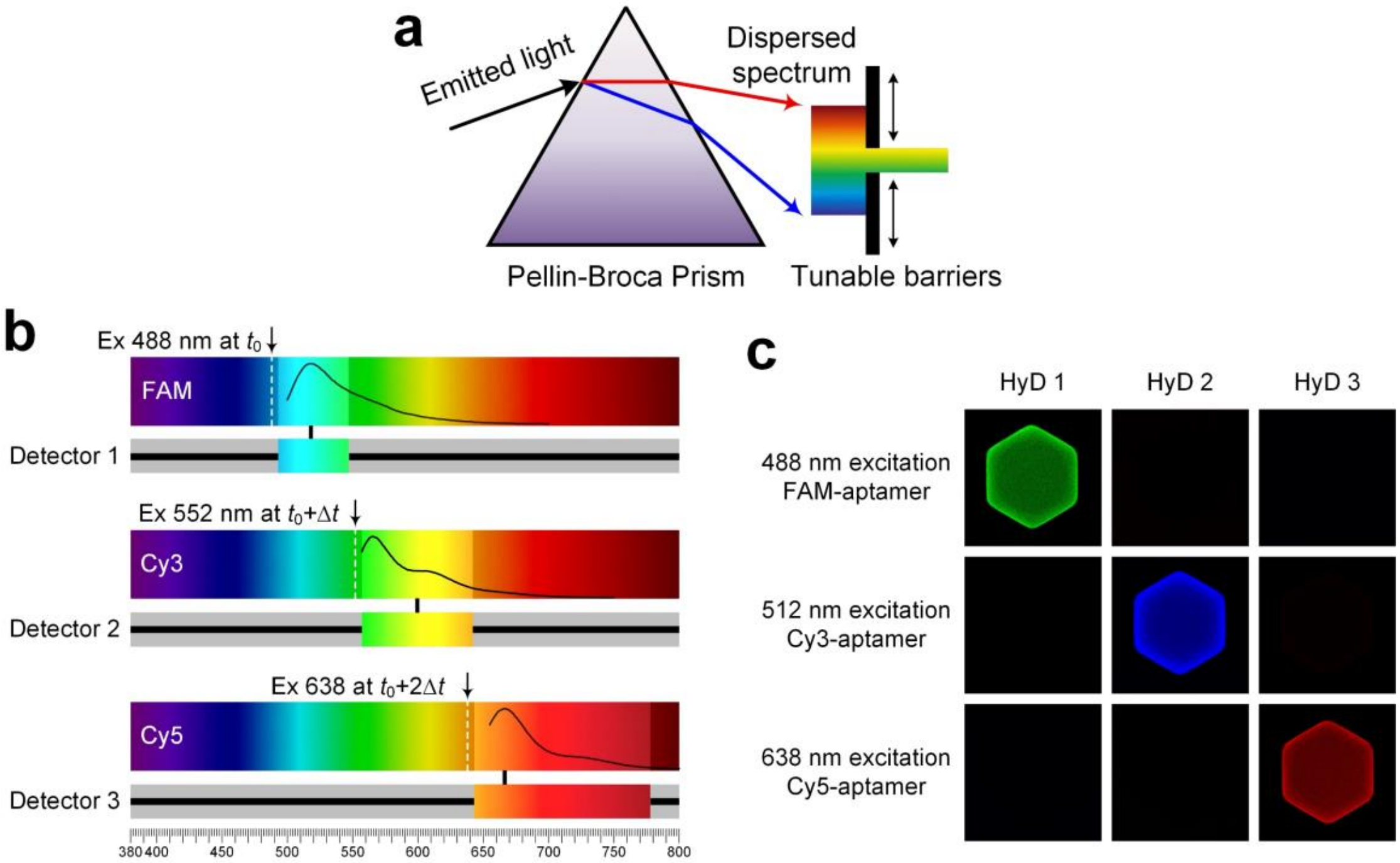

Fig. S7. (**a**) Schematic diagram illustrating the principle of spectral range selection in the Leica SP8 detector. (**b**) Detector and imaging settings used for multiplex aptamer-based fluorescence detection. (**c**) Representative fluorescence images from crosstalk evaluation, in which each excitation laser was turned on individually and all detection channels were simultaneously opened to record the fluorescence signals. The side length of the hexagonal pattern is 600 µm.

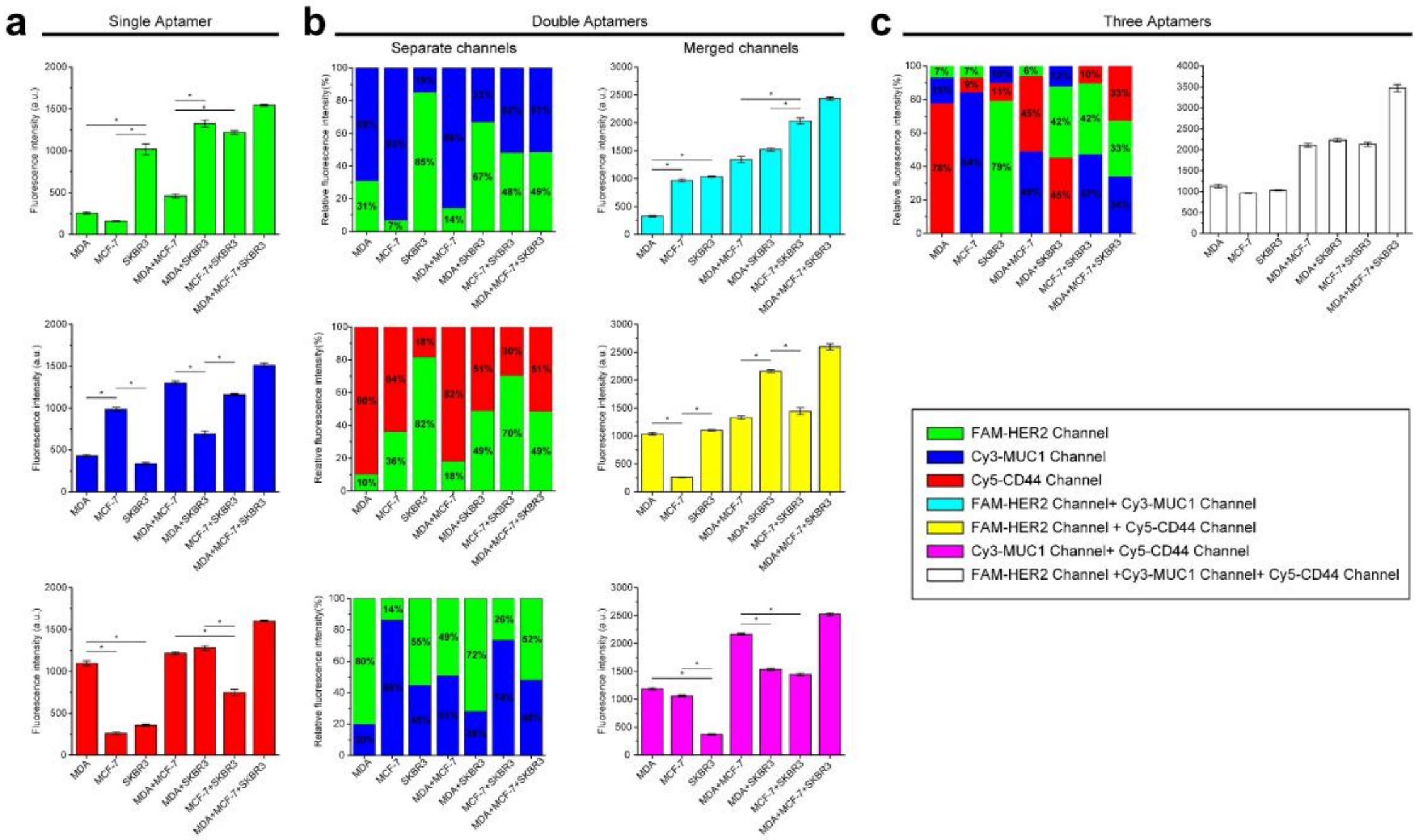

Fig. S8. (**a**) Quantitative analysis of the fluorescence intensity of each droplet spot in the single-aptamer section. (**b**) Quantitative analysis of the fluorescence intensities and relative fluorescence channel ratios of each droplet spot in the dual-aptamer section. (c) Quantitative analysis of the fluorescence intensities and relative fluorescence channel ratios of each droplet spot in the three-aptamer section. Data presented as mean ± SEM, $n = 3$. * presents the p-value < 0.05.

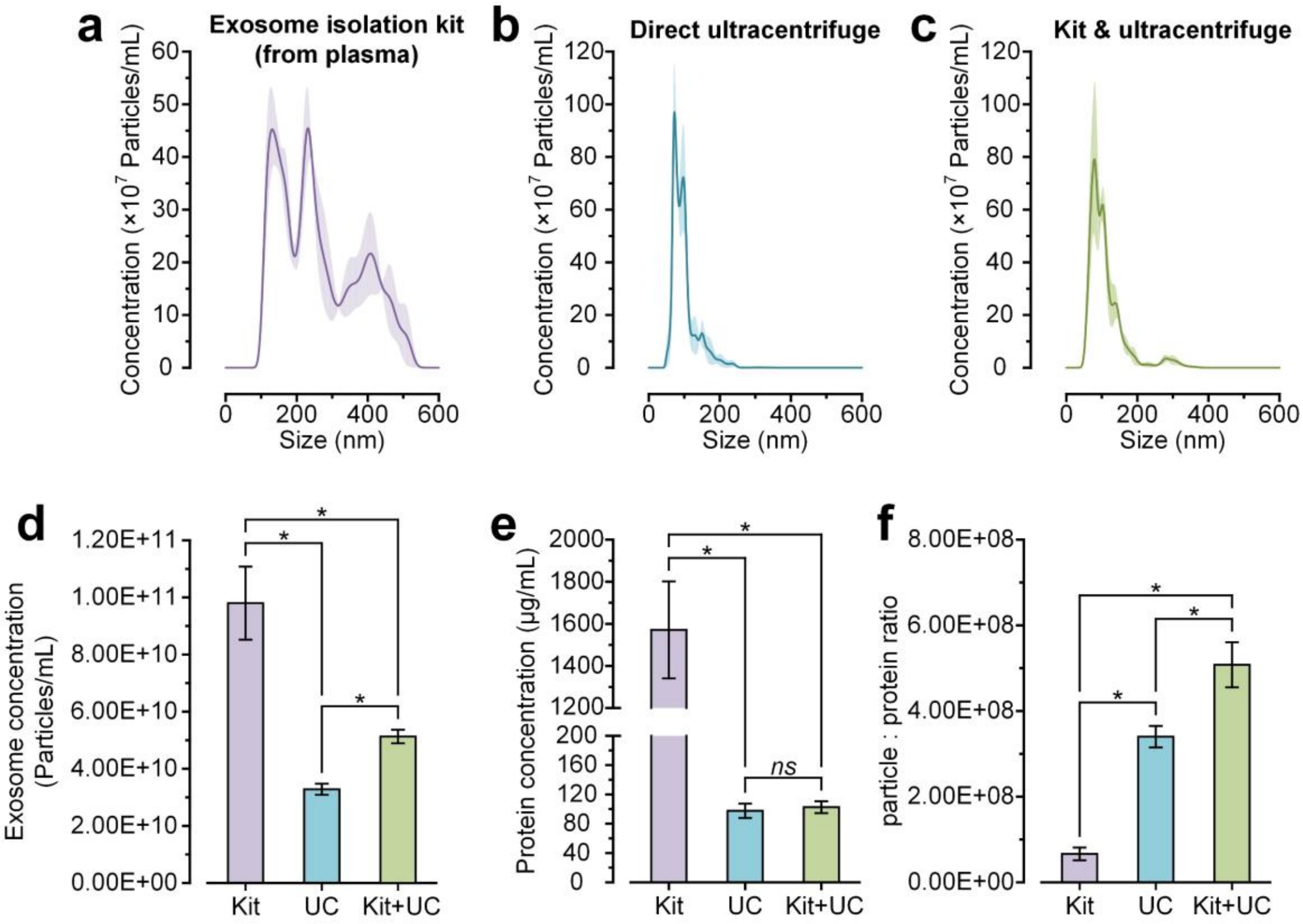

Fig. S9. (**a**)-(**c**) Representative nanoparticle tracking analysis (NTA) results of plasma exosomes from patients isolated by three different methods: kit-based isolation, ultracentrifugation, and kit-based isolation combined with ultracentrifugation. (**d**) Summary of exosome concentrations obtained by the different isolation methods. (**e**) Protein concentrations of plasma exosomes isolated by each method. (**f**) Particle-to-protein ratios calculated for plasma exosomes isolated using the different methods. Data presented as mean ± SEM, $n = 3$. * presents the p-value < 0.05.

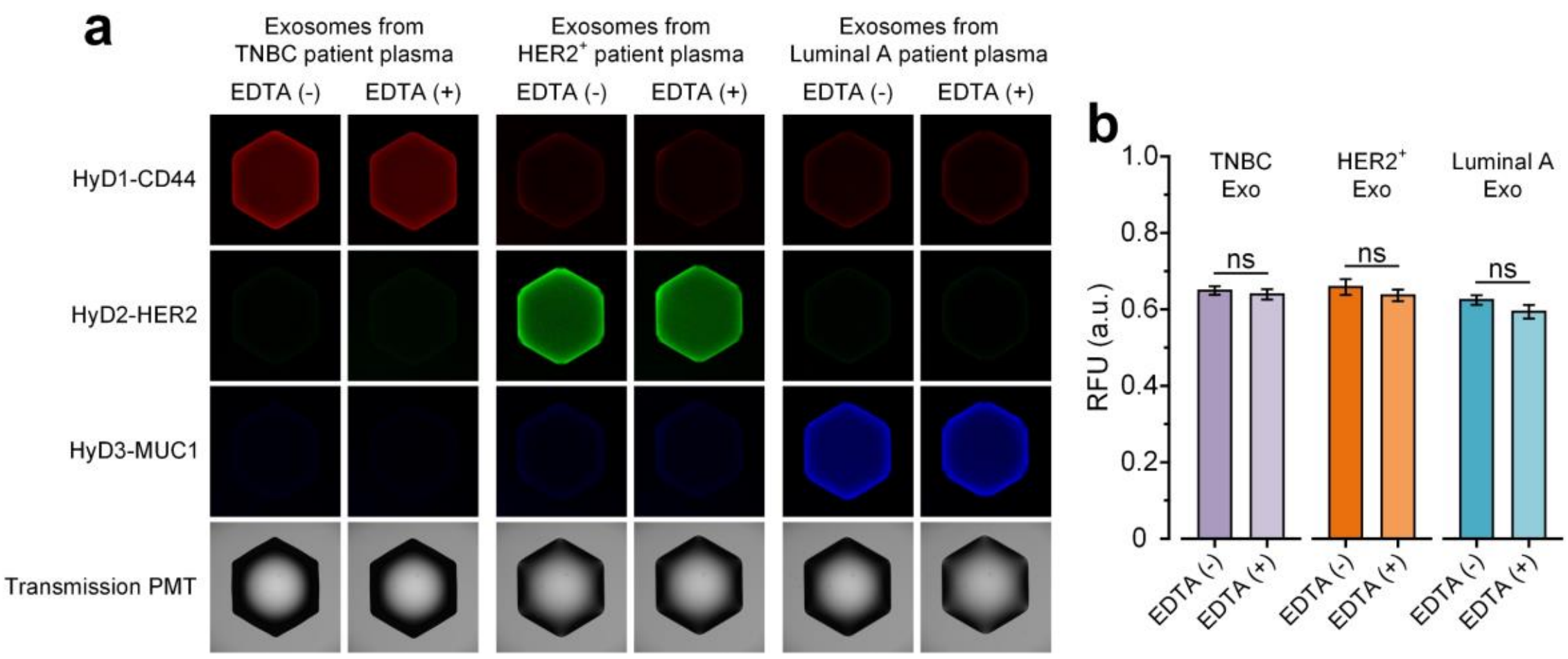

Fig. S10. (**a**) Representative fluorescence images of droplet arrays for detecting plasma exosomes isolated from patients with the triple-negative, HER2-positive, and Luminal A breast cancer subtypes, with or without EDTA treatment. No exogenous DNase I was included in this assay. The side length of the hexagonal pattern is 600 μm. (**b**) Quantitative analysis of the relative fluorescence intensities of droplets corresponding to plasma exosomes from the triple-negative, HER2-positive, and Luminal A subtypes under the indicated conditions. Data presented as mean ± SEM, n = 3, ns presents not significant.

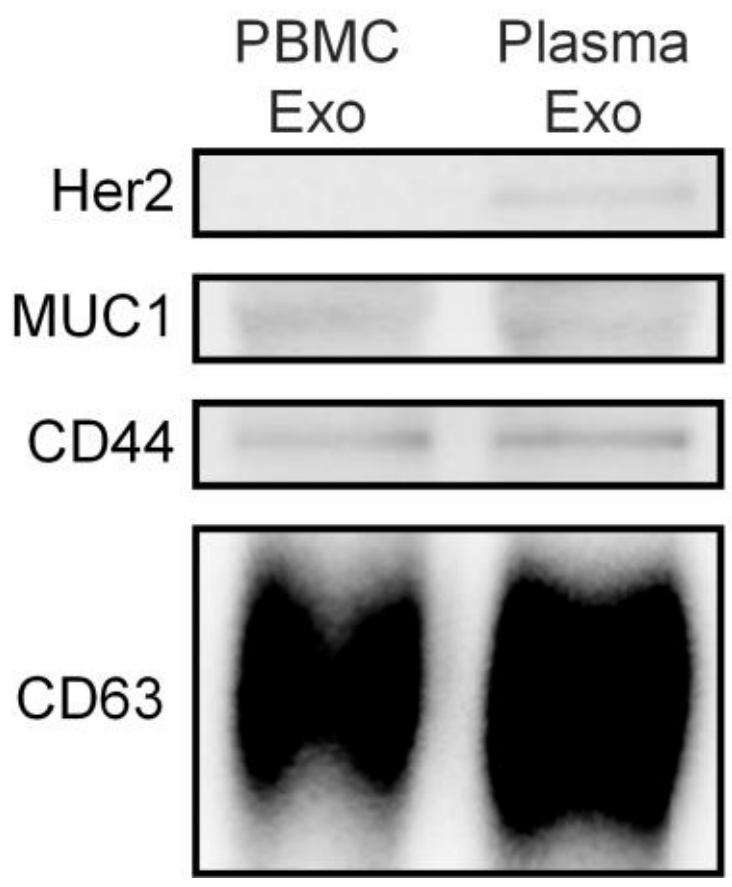

Fig. S11. Western blot analysis of the exosomal markers HER2, MUC1, CD44, and CD63 in exosomes isolated from PBMCs and plasma samples of healthy donors.

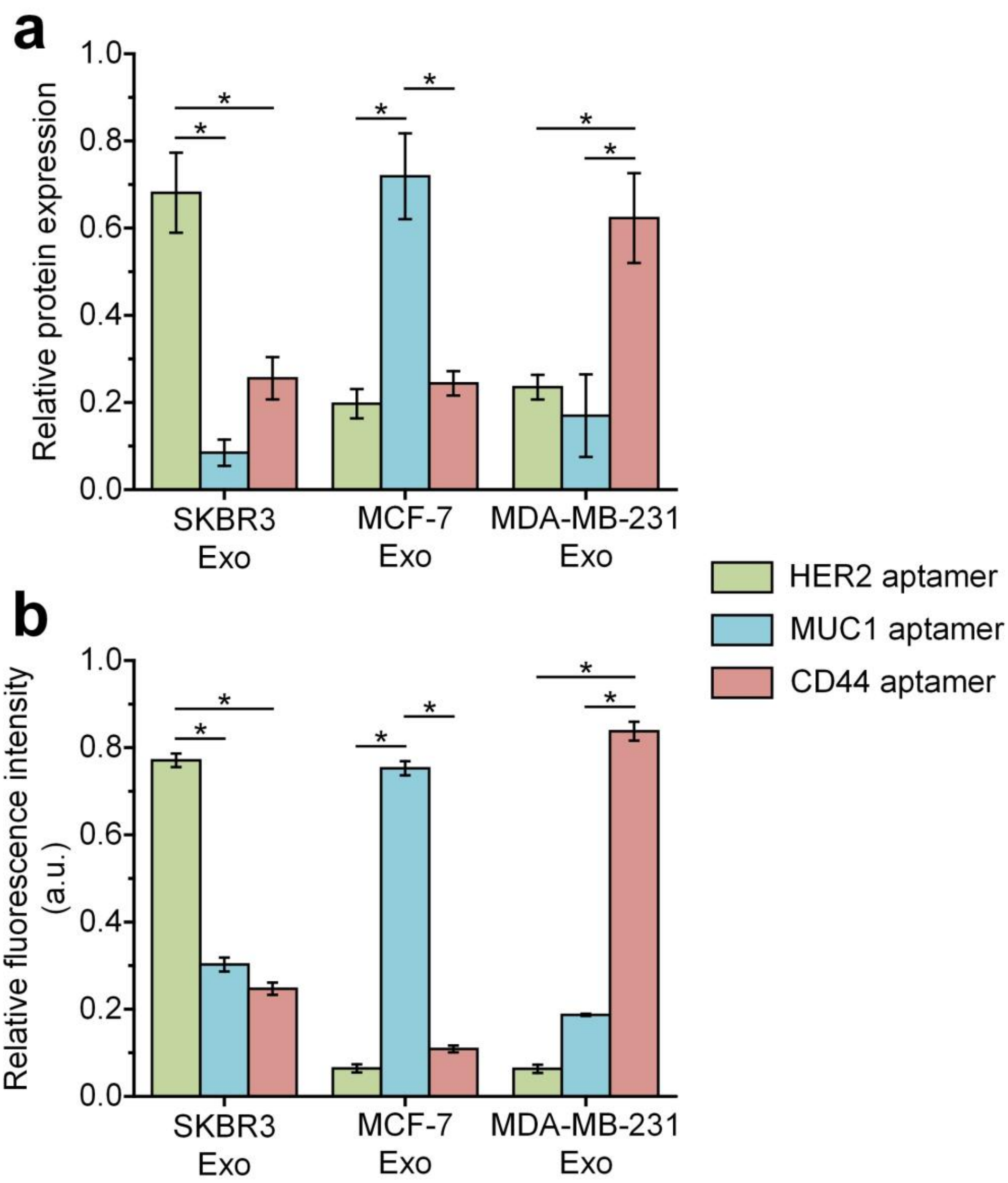

Fig. S12. Summary bar charts of the western blot analysis shown in **Fig. 2d** and the relative fluorescence intensity shown in **Fig. 2i**. Data presented as mean ± SEM, n = 3. * presents the p-value < 0.05.

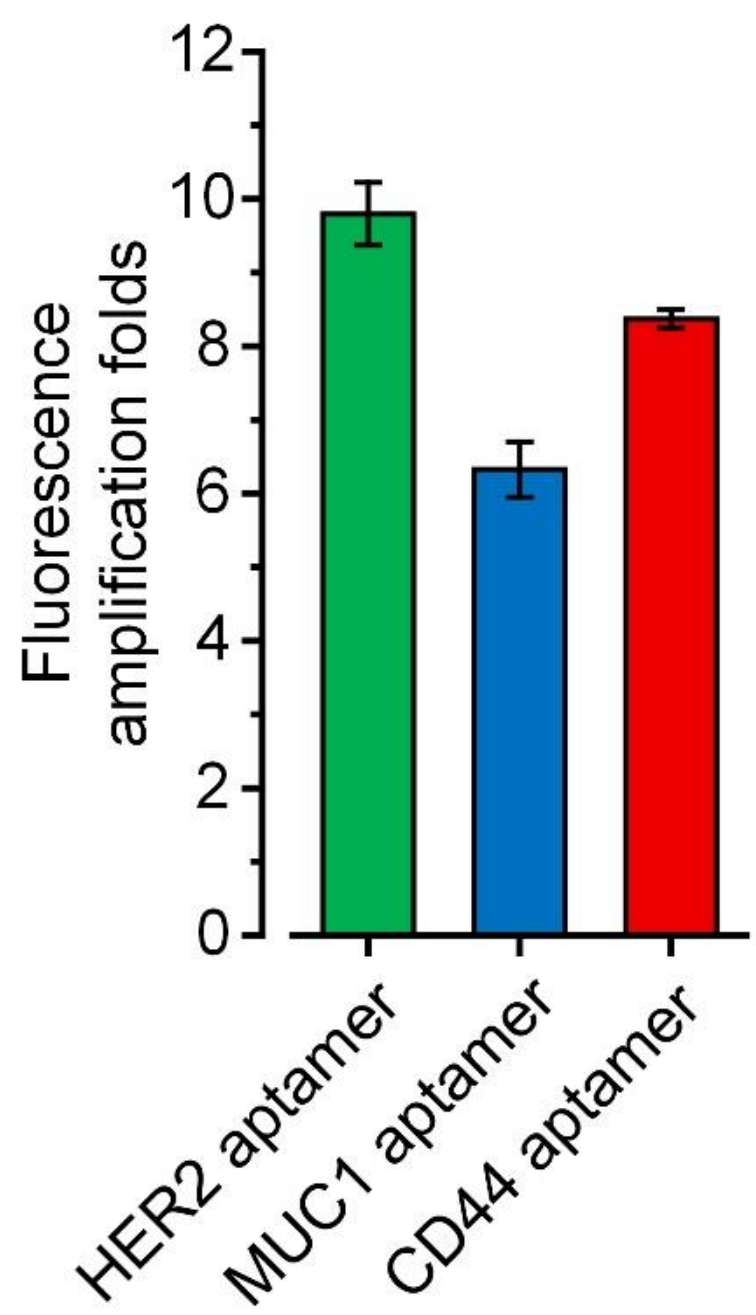

Fig. S13. Summary bar charts of the fluorescence amplification fold for each aptamer after DNase I addition. Data presented as mean ± SEM, $n = 3$.

Table S1 Information of participants in the clinical study (Cohort in Fig. 4)

Basic Information				IHC			
Tag	Gender	Age	Pathology	ER	PR	HER2 (CerbB2)	Ki-67
1	Female	27	Health control	NA	NA	NA	NA
2	Male	31	Health control	NA	NA	NA	NA
3	Male	56	Health control	NA	NA	NA	NA
4	Female	30	Health control	NA	NA	NA	NA
5	Female	49	Health control	NA	NA	NA	NA
6	Male	34	Health control	NA	NA	NA	NA
7	Female	55	Hepatic cancer	NA	NA	NA	NA
8	Male	51	Hepatic cancer	NA	NA	NA	NA
9	Male	51	Hepatic cancer	NA	NA	NA	NA
10	Male	48	Hepatic cancer	NA	NA	NA	NA
11	Female	85	Hepatic cancer	NA	NA	NA	NA
12	Male	61	Hepatic cancer	NA	NA	NA	NA
13	Female	54	Rectal cancer	NA	NA	NA	NA
14	Female	58	Rectal cancer	NA	NA	NA	NA
15	Female	75	Rectal cancer	NA	NA	NA	NA
16	Male	61	Rectal cancer	NA	NA	NA	NA
17	Male	51	Rectal cancer	NA	NA	NA	NA
18	Male	49	Rectal cancer	NA	NA	NA	NA
19	Female	57	Colon cancer	NA	NA	NA	NA
20	Male	59	Colon cancer	NA	NA	NA	NA
21	Female	50	Colon cancer	NA	NA	NA	NA
22	Male	60	Colon cancer	NA	NA	NA	NA
23	Male	50	Colon cancer	NA	NA	NA	NA
24	Male	52	Colon cancer	NA	NA	NA	NA
25	Female	40	Breast Cancer	3+, 98%	1+, <1%	1+	<1%
26	Female	58	Breast Cancer	3+, 60%	1+, <1%	0	<5%
27	Female	56	Breast Cancer	3+, 95%	1+, 2%	1+	10%
28	Female	61	Breast Cancer	3+, 95%	3+, 95%	1+	10%
29	Female	45	Breast Cancer	3+, 95%	3+, 95%	0	10%
30	Female	53	Breast Cancer	3+, 70%	3+, 30%	1+	15%
31	Female	44	Breast Cancer	3+, 100%	3+, 30%	1+	10%
32	Female	69	Breast Cancer	3+, 40%	0, 0%	1+	15%

33	Female	65	Breast Cancer	3+, 70%	1+, <1%	1+	8%
34	Female	61	Breast Cancer	3+, 95%	3+, 70%	0	15%
35	Female	40	Breast Cancer	3+, 85%	2+, 60%	0	10%
36	Female	69	Breast Cancer	3+, 95%	3+, 95%	1+	10%
37	Female	43	Breast Cancer	0, 0%	0, 0%	3+	30%
38	Female	64	Breast Cancer	0, 0%	0, 0%	2+	60%
39	Female	43	Breast Cancer	0, 0%	0, 0%	2+	30%
40	Female	61	Breast Cancer	0, 0%	0, 0%	3+	10%
41	Female	27	Breast Cancer	0, 0%	0, 0%	3+	30%
42	Female	55	Breast Cancer	0, 0%	0, 0%	3+	40%
43	Female	45	Breast Cancer	0, 0%	0, 0%	3+	80%
44	Female	43	Breast Cancer	0, 0%	0, 0%	3+	30%
45	Female	56	Breast Cancer	0, 0%	0, 0%	3+	30%
46	Female	50	Breast Cancer	0, 0%	0, 0%	2+	30%
47	Female	53	Breast Cancer	0, 0%	0, 0%	1+	50%
48	Female	52	Breast Cancer	0, 0%	0, 0%	0	75%
49	Female	42	Breast Cancer	0, 0%	0, 0%	1+	40%
50	Female	51	Breast Cancer	0, 0%	0, 0%	0	10%
51	Female	43	Breast Cancer	0, 0%	0, 0%	0	30%
52	Female	52	Breast Cancer	0, 0%	0, 0%	0	10%
53	Female	55	Breast Cancer	0, 0%	0, 0%	0	70%
54	Female	55	Breast Cancer	0, 0%	0, 0%	0	70%

Table S2 Information of participants in the clinical study (Cohort in Fig. 5)

Basic Information				IHC				
**Tag**	**Gender**	**Age**	**Pathology (Subtype)**	**ER**	**PR**	**HER2**	**FISH**	**Ki-67**
LA01	Female	42	Breast cancer (Luminal A)	3+, 95%	3+, 5%	2+	HER2 no amplification	<5%
LA02	Female	69	Breast cancer (Luminal A)	3+, 30%; 2+, 50%	3+, 20%; 2+, 15%	2+	HER2 no amplification	15%
LA03	Female	68	Breast cancer (Luminal A)	3+, >95%	3+, 70%	2+	HER2 no amplification	10%
LA04	Female	64	Breast cancer (Luminal A)	3+, 100%	3+	1+	NA	15%
LA05	Female	45	Breast cancer (Luminal A)	3+, 80%; 2+, 15%	3+, 50%; 2+, 10%	1+	NA	15%
LA06	Female	51	Breast cancer (Luminal A)	3+, 99%	3+, 10%; 2+, 20%	2+	HER2 no amplification	10%
LA07	Female	38	Breast cancer (Luminal A)	3+, 90%	3+, 30%	2+	HER2 no amplification	15%
LA08	Female	59	Breast cancer (Luminal A)	3+, 60%	1+, <1%	1+	NA	10%
LA09	Female	62	Breast cancer (Luminal A)	3+, 90%	3+, 90%	0	NA	10%
LA10	Female	67	Breast cancer (Luminal A)	3+	3+	0	NA	<5%
LA11	Female	38	Breast cancer (Luminal A)	3+	3+	0	NA	10%
LA12	Female	69	Breast cancer (Luminal A)	3+	3+	0	NA	<5%
H01	Female	46	Breast cancer (HER2 positive)	0, 0%	0, 0%	3+	NA	80%
H02	Female	52	Breast cancer (HER2 positive)	1+, <1%	0, 0%	2+	HER2 amplification	70%
H03	Female	75	Breast cancer (HER2 positive)	0, 0%	1+, <1%	2+	HER2 amplification	60%
H04	Female	38	Breast cancer (HER2 positive)	0, 0%	1+, 2%	3+	NA	30%
H05	Female	54	Breast cancer (HER2 positive)	0, 0%	0, 0%	3+	NA	30%
H06	Female	41	Breast cancer (HER2 positive)	0, 0%	0, 0%	3+	HER2 amplification	20%
H07	Female	57	Breast cancer (HER2 positive)	0, 0%	0, 0%	3+	NA	60%
H08	Female	69	Breast cancer (HER2 positive)	0, 0%	1+, <1%	2+	HER2 amplification	65%

H09	Female	72	Breast cancer (HER2 positive)	1+, <1%	0, 0%	3+	HER2 amplification	80%
H10	Female	40	Breast cancer (HER2 positive)	0, 0%	1+, 2%	2+	HER2 amplification	70%
TN01	Female	51	Breast cancer (Triple negative)	0, 0%	0, 0%	0	NA	15%
TN02	Female	50	Breast cancer (Triple negative)	0, 0%	0, 0%	2+	HER2 no amplification	60%
TN03	Female	67	Breast cancer (Triple negative)	0, 0%	0, 0%	2+	HER2 no amplification	70%
TN04	Female	60	Breast cancer (Triple negative)	0, 0%	0, 0%	1+	NA	30%
TN05	Female	68	Breast cancer (Triple negative)	0, 0%	0, 0%	1+	NA	80%
TN06	Female	53	Breast cancer (Triple negative)	1+, <1%	0, 0%	1+	NA	15%
TN07	Female	80	Breast cancer (Triple negative)	0, 0%	1+, <1%	0	NA	15%
TN08	Female	46	Breast cancer (Triple negative)	0, 0%	0, 0%	0	NA	70%
